# Warming is Associated With More Encoded Antimicrobial Resistance Genes and Transcriptions Within Five Drug Classes in Soil Bacteria: A Case Study and Synthesis

**DOI:** 10.1111/1462-2920.70097

**Published:** 2025-04-22

**Authors:** Melanie T. Hacopian, Alberto Barrón‐Sandoval, Adriana L. Romero‐Olivares, Renaud Berlemont, Kathleen K. Treseder

**Affiliations:** ^1^ Department of Ecology and Evolutionary Biology University of California, Irvine Irvine California USA; ^2^ Department of Biology New Mexico State University Las Cruces New Mexico USA; ^3^ Department of Biological Sciences California State University, Long Beach Long Beach California USA

**Keywords:** AMR, antimicrobial resistance, climate change, environmental resistance, global warming, soil bacteria, soil resistome

## Abstract

The effect of warming on anti‐microbial resistance (AMR) genes in the environment has critical implications for public health but is little studied. We collected published soil bacterial genomes from the BV‐BRC database and tested the correlation between reported optimal growth temperature and the number of encoded AMR genes. Furthermore, we tested the relationship between temperature and AMR gene transcription in a natural ecosystem by analysing soil transcriptomes from a warming manipulation experiment in an Alaskan boreal forest. We hypothesised that there is a positive relationship between warming and AMR prevalence in gene content in bacterial genomes and transcriptomic sequences, and that this effect would vary by drug class. Regarding the bacterial genomes, we found a positive relationship between the fraction of encoded AMR genes and the reported optimal temperature of soil bacteria. The drug classes tetracycline and lincosamide/macrolide/streptogramin had the strongest positive relationship with reported optimal temperature. For the case study in a natural ecosystem, we found 61 significantly upregulated AMR gene‐associated transcripts spanning eight drug classes in warmed plots. In the Alaskan soil samples, we found that warming elicited the strongest positive effect on transcripts targeting lincosamide/streptogramin, beta‐lactam and phenicol/quinolone antibiotics. Overall, higher temperatures were linked to AMR gene prevalence.

## Graphical abstract

We found a positive relationship between reported optimal temperature and the number of encoded AMR genes in published soil bacterial genomes. We also observed increased AMR transcription in soil transcriptomes undergoing experimental warming and drying in a natural ecosystem. Warming effects varied by drug class.

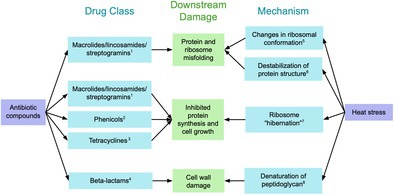

## 1 Introduction

Recently, investigators have documented a relationship between increased temperatures and antimicrobial resistance (AMR) acquisition and expression under laboratory conditions (Cruz‐Loya et al. 2019; Rodríguez‐Verdugo et al. 2020). These findings hint that human‐caused warming might increase the prevalence of AMR, with potential consequences for public health. Yet, studies testing the connection between temperature and AMR gene presence and regulation are rare, with only a handful being performed in natural ecosystems (Donhauser et al. 2020; Li, Liu, et al. 2022; MacFadden et al. 2018; Wang et al. 2024). Recent evidence suggests increases in ambient temperature increase the prevalence of resistant strains of bacteria (Li, Sun, et al. 2022; Li et al. 2023; McGough et al. 2020). Examining the relationship between warming and AMR in situ in soil is critical, since soil acts as a reservoir of AMR genes (Do et al. 2023; Yang et al. 2020). Here, we addressed this knowledge gap first by examining publicly available bacterial genomes that were isolated from soil to investigate the correlation between reported optimal growth temperature and the number of encoded AMR genes. Since possessing an AMR gene does not guarantee that the gene is transcribed, we then evaluated the effect of warming on AMR transcript abundance in soil samples collected from a relatively remote Alaskan boreal forest.

Bacteria may have co‐opted heat stress genes to develop resistance to antibiotics (Cruz‐Loya et al. 2019). Furthermore, it has been hypothesised that high temperatures and some antimicrobial compounds affect microbial cells similarly and elicit overlapping responses (Cardoso et al. 2010; Rodríguez‐Verdugo et al. 2020). High temperatures damage bacterial cells by causing protein unfolding and damage to DNA/RNA, causing the heat‐shock response (Lindquist 1986; Richter et al. 2010). The heat‐shock response, which is highly conserved across all domains of life, triggers the upregulation of protein chaperones and proteases that mitigate the damage caused by protein unfolding. Similar to heat stress, antibiotic compounds inhibit key cellular processes, including DNA/RNA and protein synthesis (Gottlieb and Shaw 2013). Both stressors can also damage cells by weakening the cell wall (Kohanski et al. 2008; Teixeira et al. 1997; Thackray and Moir 2003; Zhang et al. 2023). In response to antibiotics, bacteria may become tolerant or fully resistant by slowing their growth, genetically mutating the drug target, deactivating the drug with an enzyme, or exporting the harmful compound via an efflux pump (Brauner et al. 2016; Kester and Fortune 2014). In addition, genes involved in the heat shock response may have evolved before bacteria were exposed to the first antimicrobial compounds (Dcosta et al. 2011; Lindquist 1986; Schwartzman and Lineweaver 2004; Stetter 2006). This suggests that microbes may evolve resistance to antimicrobial compounds by altering existing machinery in addition to creating entirely new genes and pathways (Dragosits et al. 2013; Święciło 2016). Previous lab‐based studies have documented overlapping physiological effects of temperature and selected drugs in *Escherichia coli* (Cardoso et al. 2010; Cruz‐Loya et al. 2019; Rodríguez‐Verdugo et al. 2020; VanBogelen and Neidhardt 1990). In one study, *E. coli* acquired resistance to rifampicin when adapted from 37.0°C to 42.2°C (Rodríguez‐Verdugo et al. 2013). Additionally, antibiotic stress shifted the optimal growth temperatures of *E. coli* , possibly explained by the similar downstream damage imposed by antibiotics and temperature (Cruz‐Loya et al. 2021). Altogether, physiological responses to increased temperature may spur the acquisition of AMR genes.

The relationship between AMR and warming has been primarily tested in clinical settings or laboratory experiments on single species (Cardoso et al. 2010; Poole 2014; Rodríguez‐Verdugo et al. 2013; Yi et al. 2024). However, as land surface temperatures are expected to rise due to climate change, we ask how increased temperatures might promote the emergence of AMR bacteria in environmental systems (Cayan et al. 2010; Cook et al. 2015). Previous experiments have documented resistance genes in natural ecosystems (Kaba et al. 2020; Lovero and Mota‐Bravo 2022; Martiny et al. 2011; McGough et al. 2020; Sayah et al. 2005; Seyfried et al. 2010; Wang et al. 2024), but few have examined a link to temperature.

We must understand this connection to determine if warming might exacerbate the spread of AMR. The extensive use of antibiotics in medicine and agriculture has selected for AMR in bacteria (Li et al. 2009; Martinez 2009). The resistant strains can enter the soil, forming a reservoir of AMR genes (Rodríguez‐Verdugo et al. 2020). Then horizontal gene transfer (HGT)—which may be more frequent at increased temperature—can propagate resistance machinery to antimicrobial compounds from one strain to another (Fuchsman et al. 2017; Kamal et al. 2021; Walsh et al. 2011; Yang et al. 2020). At the same time, AMR can develop naturally in soils as bacteria release antimicrobial compounds targeting one another (Mullis et al. 2019). Moreover, many bacterial infections of humans and animals are contracted from the environment, including soil (Bandyopadhyay and Samanta 2020; Baumgardner 2012; Burmølle et al. 2010). If warming increases the content and subsequent expression of AMR genes in the soil, the exposure of humans and animals to AMR strains may rise.

We first conducted a bioinformatic synthesis in which we evaluated AMR genes detected in bacterial genomes isolated from soil and available on the BV‐BRC database (Olson et al. 2023). In addition to gene content, we evaluated expression by examining AMR genes detected in soil metatranscriptomes undergoing long‐term warming. We hypothesised a positive relationship between higher temperatures and AMR prevalence (Hypothesis 1). For the synthesis, we asked: is there a relationship between the reported optimal growth temperature of bacteria and the number of AMR genes encoded within their genomes? Consequently, we predicted that bacteria with higher reported optimal temperatures will encode a greater fraction of AMR genes. For the case study, we asked what effect increased temperature has on the transcription of AMR genes in a natural setting? We predicted that warmed soil communities will have a greater number of significantly upregulated AMR gene‐associated transcripts than control communities, based on the rationale that transcription of these genes is favourable to mitigate detrimental effects of increased temperature.

Since different antimicrobial compounds have distinct physiological effects on bacterial cells, the interaction between temperature and resistance genes may differ based on which compound the resistance gene targets (Cruz‐Loya et al. 2019; VanBogelen and Neidhardt 1990). For example, genes that confer resistance to drugs affecting nucleic acids or membrane functionality might be favoured under increased temperature (Loughman et al. 2016; Rodríguez‐Verdugo et al. 2013). On the contrary, the frequency and expression of AMR genes targeting drugs exhibiting little overlap with temperature adaptation would be unaffected by increased temperature. We therefore hypothesised that the effect of temperature on AMR varies by drug class (Hypothesis 2). We predicted to observe this trend in both the bioinformatic synthesis and the case study.

## 2 Methods

### 2.1 AMR Analysis of Soil Bacterial Genomes (Genome Study)

#### 2.1.1 Extracting Bacterial Genomes and AMR Gene Data From BV‐BRC

Sequenced bacterial genomes were retrieved from the BV‐BRC database (Olson et al. 2023; Wattam et al. 2017). We selected genomes with a reported optimal growth temperature. Genomes with optimal temperature given as a range were removed from analysis, as a temperature range might be indicative of a broader approximation. We also retrieved the complete taxonomy for each of the selected genomes. Next, we retrieved the predicted protein sequences for each genome and analysed them using AMRFinder (v.3.11.26) (Feldgarden et al. 2021). The AMRFinderPlus database incorporates the Pathogen Detection Reference Gene Catalogue, Pathogen Detection Reference Gene Hierarchy and Reference HMM Catalogue. The AMR reference gene catalogue includes 5588 AMR genes and 8 heat resistance genes. The AMR genes cover 31 drug classes.

#### 2.1.2 Statistical Analyses for Bioinformatic Investigation

**FIGURE 1:**
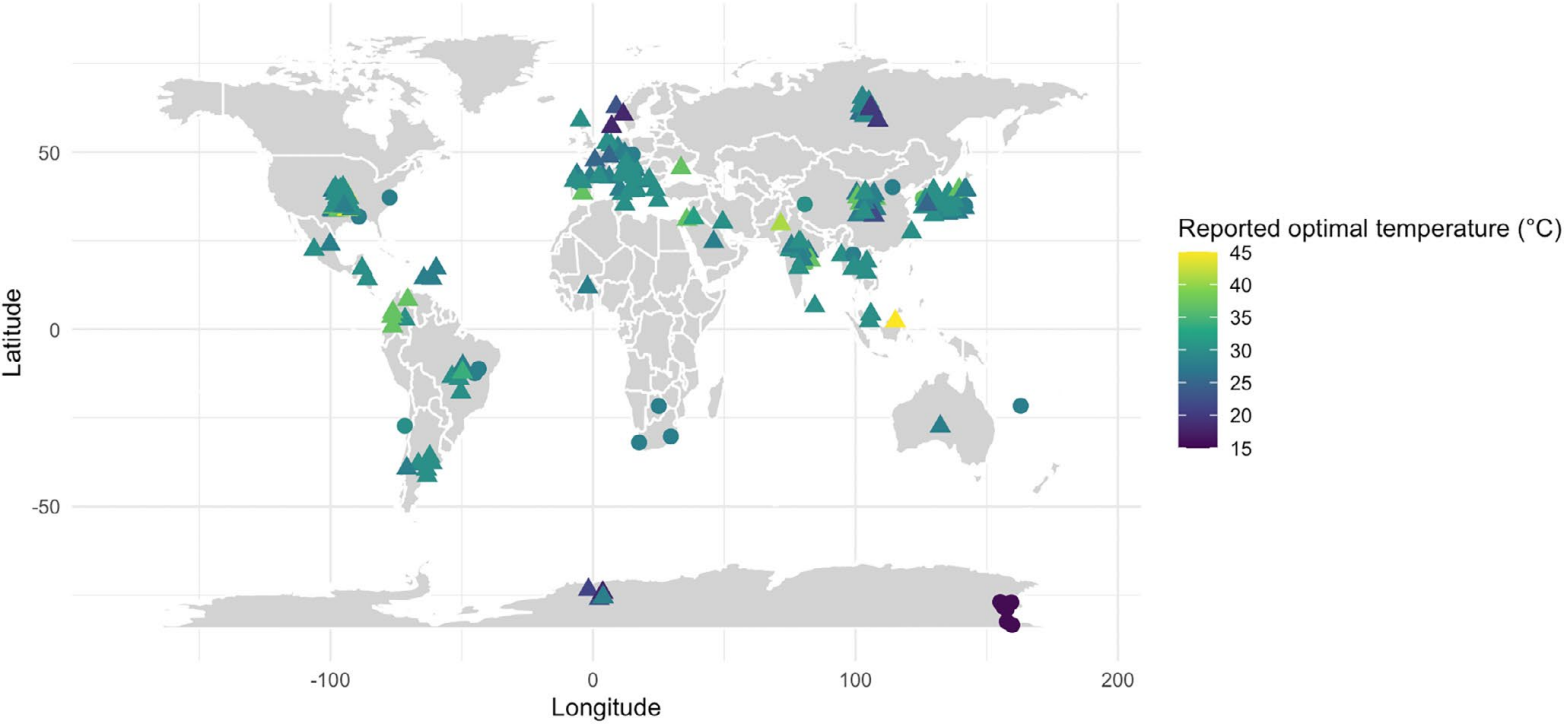
Global distribution of soil genomes used in investigation of soil AMR genes (*n* = 279, across 44 countries). The colour of the symbols represents reported optimal temperature (°C). Circle‐shaped symbols indicate genomes for which exact latitude and longitude were available. Triangle‐shaped symbols represent genomes associated with a country of origin but lack precise coordinate data.

After running AMRFinder on 5087 bacterial genomes for which optimal temperature data was available, we observed 10,733 AMR gene hits. We counted the total number of hits for each genome and then normalised the values by dividing the number of hits by the number of protein coding genes (PEGs) of each genome. From these data, we kept 296 genomes that were isolated from soil based on key words provided in the ‘Isolation Source’ metadata category (e.g., ‘Soil’, ‘soil’, ‘soil sample’, etc.). Our genome dataset spanned 44 countries (Figure 1). We excluded extreme thermophiles with a reported optimal temperature > 50°C, since humans rarely interact with their natural ecosystems (Brock 1978; Wagner and Wiegel 2008). To test for a relationship between encoded AMR genes and reported optimal growth temperature, we used the ‘lm’ function from the R package ‘stats’ (v.3.6.2) (R Core Team 2013) to perform a mixed hierarchical model that accounted for the phylum and genus of isolates. In our model, reported optimal temperature was a fixed effect. Genus was a random factor nested within phylum, another random factor. The normalised AMR count was the dependent variable. We performed linear regressions for each drug class following the same formula. Since an increase in normalised AMR counts could stem from a relative decrease in other genes, we also performed regressions on non‐normalised counts. We also conducted the same tests after removing isolates with a reported optimal temperature of 37°C, as any effect on encoded AMR genes could have resulted from human exposure rather than temperature.

### 2.2 AMR Analysis of Warmed Soil Metatranscriptomes (Case Study)

#### 2.2.1 Field Site

The field site used in this study was in an Alaskan boreal forest (63°55′N, 145°44′W). It was located on undeveloped land on the Fort Greely Army Base, which abutted Delta Junction, a town with about 1000 residents. Mean monthly air temperature ranged from −20°C in January to 16°C in July, with a record high temperature of 33°C (Treseder et al. 2004). Soil pH is 4.9 (Allison and Treseder 2008). The forest was dominated by *Picea mariana* (black spruce). The warming manipulation experiment was initialised in 2005 as described in Allison and Treseder (2008). In brief, greenhouses were erected along with neighbouring control plots to establish warming (*n* = 4) and control (*n* = 4) treatments. During the growing season (from May to September), the air in the greenhouses averaged 1.6°C higher than that in the control plots. Likewise, the greenhouse soil (at 5 cm depth) was 0.5°C warmer than control soil. Historically, warming increased the average soil temperature from 9.3°C to 10.1°C with increases in daily maximum, minimum and mean temperature (Allison and Treseder 2008). Between 2005 and 2007, soil temperature reached a maximum of 22.0°C in warmed plots compared with 19.8°C in control plots. Increased temperature led to higher evapotranspiration in the greenhouses, reducing soil moisture by an average of 22%. In addition, warming decreased PO_4_^3−^ availability and bacterial abundance. Warming increased NH_4_^+^ and NO_3_^−^ availability (Allison and Treseder 2008).

#### 2.2.2 Sample Collection and Sequencing

Sample collection, RNA extraction and sequencing is described in detail in Romero‐Olivares et al. (2019). Sampling was carried out on July 4, 2015. On that day, the maximum air temperature was 24.4°C and the average air temperature was 16.7°C (Delta Junction area, National Oceanic and Atmospheric Administration (NOAA) weather service). There was no precipitation on that day. Briefly, 1 g of homogenised soil from each plot was collected and soaked in 5 mL of Qiagen Lifeguard Soil Preservation Solution. Samples were kept on ice for transfer to a −80°C freezer and extracted within 1 week of collection. RNA was extracted using the Qiagen PowerSoil kit. High quality samples were sequenced by the Joint Genome Institute (JGI) using the Illumina Hiseq 2500 system. Sequencing results can be found in JGI's data portal under the following project IDs: 1107‐496, 1107‐499, 1107‐504, 1107‐507, 1107‐509, 1107‐514, 1107‐519 and 1107‐520.

#### 2.2.3 Extracting AMR Transcripts From Soil Transcriptome Data

Raw reads from all eight plots were downloaded from JGI's data portal. Adapters were trimmed with Trimmomatic (v.0.39) using Illumina TruSeq3‐PE adapters, and any reads < 36 bp were removed (Bolger et al. 2014). Quality of trimming was checked with fastQC (v.0.11.09) before moving on to rRNA filtering (Andrews 2010). rRNA was removed using sortmeRNA (v.4.3.6) and SILVA databases containing prokaryotic 16s and 28s and eukaryotic 18s, 28s, 5s and 5.8s rRNA reference sequences (Kopylova et al. 2012). The sorted mRNA reads were then combined to assemble a de novo metatranscriptome using Megahit (v.1.2.9) (Li et al. 2015). To predict protein‐coding genes within the metatranscriptome we used Prodigal (v.2.6.3) (Hyatt et al. 2010). Finally, to identify AMR genes and heat resistance genes, the protein output of Prodigal for each treatment was blasted against the AMRFinderPlus database using AMRFinder.

To quantify transcript abundance, we mapped the trimmed mRNA short reads back to a reference index produced from the predicted gene sequences from the reference metatranscriptome using Salmon (v.1.8.0) (Patro et al. 2018). Briefly, Salmon is a pseudo‐alignment tool that estimates transcript level abundances from RNA sequence data while accounting for sequence‐specific, GC content and positional biases. Salmon returned quantification files for each sequenced dataset, including the estimate of the number of reads mapping to each transcript and the relative abundance of transcripts in transcript per million (TPM).

#### 2.2.4 Extracting Taxonomy From Soil Transcriptome Data

We explored shifts in bacterial community composition as one possible mechanism underlying shifts in AMR transcription. To classify the taxonomy of reads we used the taxonomic sequence classifier Kraken2 (v.2.1.2) and the Greengenes (v.13.5) 16s rRNA reference database on the previously separated rRNA short reads (DeSantis et al. 2006; Wood et al. 2019). To measure the abundance of taxa in each treatment by taxonomic rank, we used Bracken (v.2.8) and the previously generated taxonomy report from Kraken2. For phylum and genera analyses, we kept only the top 15 most abundant phyla and genera found in each treatment. We calculated relative abundance by grouping the taxonomic ranks by treatment, followed by dividing the fraction of total reads for each rank by the sum of the fraction of total reads and multiplying by 100. We generated relative abundance plots using ggplot2 (v.3.4.4) in R (v.4.2.1) (Dixon 2003; Wickham 2011; Wickham et al. 2019).

#### 2.2.5 Statistical Analyses for Soil Transcriptome Case Study

To examine differently transcribed genes, we used the DESeq2 (v.1.36.0) package in R (Love et al. 2014). To prepare the count data from Salmon for DESeq2, we focused on transcripts detected at least 50 times across samples, since more abundant transcripts are more likely to show biologically meaningful differences between treatments. This left us with a sample size of 112,226 highly expressed transcripts. Next, we used DESeq2 to measure differential expression of transcripts between the control and warmed samples. DESeq2 calculated log2 fold changes and Wald‐test *p* values adjusted using the Benjamini and Hochberg method. To identify significantly upregulated AMR and heat resistance genes, we merged data sets of the DESeq2 results and AMRFinder output using the innerjoin function provided by the tidyverse package (v.2.0.0) in R (Wickham et al. 2019).

To measure TPM changes between the control and warmed treatment, we used the TPM data from the Salmon results. We summed the TPM values for each of the 35 unique drug classes. We then performed a MANOVA in Systat 13 in which our dependent variables were the summed TPM values for each drug class, and our independent variable was treatment. Then, to assess the different magnitudes by which specific drug classes are affected by warming, we calculated the Cohen's *D* effect size for each drug class by taking the difference between the means of the warmed and control groups and dividing that value by the pooled standard deviation of both groups. A positive Cohen's *D* value indicates a greater expression in the warmed group compared with the control. A larger value of Cohen's *D* indicates a larger difference between the groups. The common benchmarks for interpreting the Cohen's *D* values are ~0.2 for a small effect, ~0.5 for a medium effect and ~0.8 or greater for a large effect (Cohen 1988; Lakens 2013).

To measure relative abundance changes of bacterial phyla and genera between control and warmed groups, we tested the effect of treatment on relative abundance using the Wilcoxon signed‐rank test and adjusted *p* values using the Benjamini and Hochberg method. We used the ‘wilcox.test’ function provided by the ‘stats’ (v.3.6.2) package in R (Bauer 1972). Code for this study, in addition to identifiers of genomes extracted from the BV‐BRC, is deposited at https://github.com/melaniehacopian/AMR_workflow.

## 3 Results

### 3.1 AMR Analysis of Soil Bacterial Genomes (Genome Study)

**FIGURE 2:**
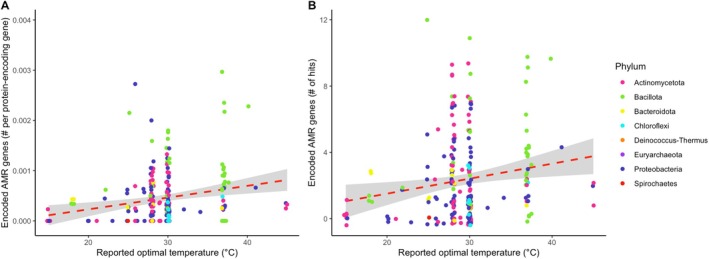
Positive relationship between reported optimal growth temperature and (A) the number of encoded AMR genes normalised by the number of protein‐encoding genes (PEGs) or (B) the number of total encoded AMR genes. Each symbol represents a bacterial genome isolated from soil (*n* = 280 genomes). Colour represents phylum.

A regression analysis on 280 genomes derived from soil showed that reported optimal growth temperature was positively correlated with both the normalised fraction of encoded AMR genes (Figure 2, conditional *R* = 0.55, *p* = 0.025) and the total number of genes (*R* = 0.61, *p* = 0.009). When isolates with a reported optimal temperature of 37°C were removed from the dataset, the relationship was still significant (Figure S1, *R* = 0.59 fraction, 0.64 total, *p* < 0.05 for both). Separate regressions on each drug class revealed significant positive relationships for the drug classes aminoglycoside (Table 1, *p* = 0.047 total), beta‐lactam (*p* = 0.027 total), lincosamide/macrolide (*p* = 0.028 fraction, 0.029 total), lincosamide/macrolide/streptogramin (*p* = 0.026 fraction), phenicol (*p* = 0.020 fraction) and tetracycline (*p* = 0.020 fraction, 0.008 total). Our first and second hypotheses were supported, since soil bacteria with higher reported optimal temperatures encoded a greater number of AMR genes, and this result varied by drug class.

**TABLE 1:** Regression statistics for reported optimal growth temperature and normalised/total encoded AMR genes per different drug classes (*n* = 280 soil genomes).

Drug class	Mechanism of action	*T* (normalised/total)	Conditional *R* (normalised/total)	*p* ^a^ (normalised/total)
Beta‐Lactam	Inhibition of cell wall synthesis	0.90/2.23	0.42/0.68	0.369/**0.027**
Fosfomycin	Inhibition of cell wall synthesis	−0.18/−0.39	−0.34/−0.27	0.856/0.701
Glycopeptide	Inhibition of cell wall synthesis	1.40/0.38	0.20/0.66	0.165/0.705
Efflux	Variable	−0.26/−0.22	−0.32/−0.31	0.795/0.829
Phenicol/Quinolone	Variable	1.57/1.57	0.10/0.10	0.116/0.117
Bleomycin	Inhibition of DNA synthesis	−0.27/0.27	−0.02/−0.02	0.788/0.788
Sulfonamide	Inhibition of DNA synthesis	−0.05/−0.08	−0.06/−0.06	0.961/0.938
Rifamycin	Inhibition of RNA synthesis	1.42/0.41	0.66/0.48	0.157/0.685
Aminoglycoside	Inhibition of protein synthesis (30S)	1.20/2.01	0.08/0.34	0.230/**0.047**
Streptothricin	Inhibition of protein synthesis (30S)	−0.76/−0.97	−0.42/−0.36	0.449/0.334
Tetracenomycin	Inhibition of protein synthesis (30S)	−0.27/−0.27	−0.02/−0.02	0.788/0.788
Tetracycline	Inhibition of protein synthesis (30S)	2.25/2.69	0.50/0.37	**0.025/0.008**
Tuberactinomycin	Inhibition of protein synthesis (30S)	0.143/0.140	0.02/0.02	0.886/0.886
Lincosamide	Inhibition of protein synthesis (50S)	−0.07/−0.07	−0.10/−0.10	0.946/0.946
Lincosamide/Macrolide	Inhibition of protein synthesis (50S)	2.20/2.20	0.14/0.14	**0.028/0.029**
Lincosamide/Macrolide/Streptogramin	Inhibition of protein synthesis (50S)	2.25/1.93	0.55/0.29	**0.026**/0.055
Lincosamide/Streptogramin	Inhibition of protein synthesis (50S)	0.86/1.03	0.50/0.49	0.392/0.310
Macrolide	Inhibition of protein synthesis (50S)	−0.38/0.24	−0.76/0.77	0.706/0.811
Macrolide/Streptogramin	Inhibition of protein synthesis (50S)	1.57/1.57	0.12/0.12	0.117/0.119
Phenicol	Inhibition of protein synthesis (50S)	2.32/1.82	0.58/0.64	**0.020**/0.069
Streptogramin	Inhibition of protein synthesis (50S)	0.47/0.19	0.39/0.41	0.636/0.846
Thiostrepton	Inhibition of protein synthesis (50S)	Insufficient data		

^a^ Significant *p* values in bold.

### 3.2 AMR Analysis of Warmed Soil Metatranscriptomes (Case Study)

#### 3.2.1 Bioinformatics

Megahit assembled a reference metatranscriptome comprised of a total of 6,439,119 contigs consisting of a total of 3,277,156,939 base pairs (bp). The average contig size for the assembly was 508 bp, with N50 of 500 bp. From these contigs, prodigal predicted 7,703,022 potential genes. From the prodigal detected coding sequences, AMRFinderPlus returned 48,409 hits matching AMR genes and 4303 hits for heat resistance genes. Our Salmon index, a structure analogous to a reference metatranscriptome that Salmon uses to quasi‐map RNA‐seq reads, contained a total of 7,691,596 distinct sequences (Patro et al. 2018). In total, the control plots had an average mapping rate (i.e., the percent of transcripts that aligned to Salmon's reference metatranscriptome) of 18.82% and the warmed plots 20.86%. Kraken2 classified 11.69% of reads (18,819,567 reads) as Bacteria for the control treatment rRNA short reads, and 12.47% of reads (14,829,348 reads) for the warmed treatment. Remaining reads either belonged to non‐bacterial taxa or lacked sufficient similarity to any taxa within the reference database.

#### 3.2.2 Differential Expression

DESeq2 returned differential expression results for a dataset of 112,226 transcripts when comparing the control (*n* = 4) and warmed (*n* = 4) plots. In total, 65 AMR transcripts varied significantly between treatments. Specifically, 61 were found to be significantly upregulated in the warming treatment (Table 2, Figure S2a, *p* < 0.05, log2 fold change > 2). These results provide support for our first hypothesis, since there was a greater number of AMR transcripts that are upregulated in the warmed treatment. For heat resistance associated transcripts, 11 transcripts were significantly upregulated in the warmed treatment while 5 were downregulated (Table S1, Figure S2b).

**TABLE 2:** Significantly upregulated and downregulated AMR transcripts with average fold change values (*p* < 0.05 for all).

Gene symbol	Drug class	Mechanism of action	Sequence name	Average log2 fold change
*Upregulated*				
vanS‐Sc	Glycopeptide	Inhibition of cell wall synthesis	VanSc‐type vancomycin resistance histidine kinase VanS	21.53
vanS‐M	Glycopeptide	Inhibition of cell wall synthesis	VanM‐type vancomycin resistance histidine kinase VanS	21.26
vanS‐G	Glycopeptide	Inhibition of cell wall synthesis	VanG‐type vancomycin resistance histidine kinase VanS	21.95
vanR‐I	Glycopeptide	Inhibition of cell wall synthesis	Vancomycin resistance response regulator transcription factor VanR‐I	21.06
vanR‐D	Glycopeptide	Inhibition of cell wall synthesis	Vancomycin resistance response regulator transcription factor VanR‐D	20.77
vanY‐D	Glycopeptide	Inhibition of cell wall synthesis	Transpeptidase‐like D‐Ala‐D‐Ala carboxypeptidase VanY‐D	21.04
vanH‐D	Glycopeptide	Inhibition of cell wall synthesis	D‐lactate dehydrogenase VanH‐D	21.78
tet(57)	Tetracycline	Inhibition of protein synthesis (30S subunit)	Tetracycline efflux MFS transporter Tet(57)	21.28
tcr3	Tetracycline	Inhibition of protein synthesis (30S subunit)	Tetracycline efflux MFS transporter Tcr3	21.43
tetB(60)	Tetracycline	Inhibition of protein synthesis (30S subunit)	Tetracycline efflux ABC transporter Tet(60) subunit B	21.07
tetA(58)	Tetracycline	Inhibition of protein synthesis (30S subunit)	Tetracycline efflux ABC transporter Tet(58) subunit A	20.66
toprJ	Tetracycline	Inhibition of protein synthesis (30S subunit)	Multidrug efflux transporter outer membrane subunit TOprJ	21.13
tcmA	Tetracenomycin	Inhibition of protein synthesis (30S subunit)	Tetracenomycin C efflux MFS transporter	17.28
vgbC	Streptogramin	Inhibition of protein synthesis (50S subunit)	Streptogramin B lyase Vgb(C)	20.35
vgb(B)	Streptogramin	Inhibition of protein synthesis (50S subunit)	Streptogramin B lyase Vgb(B)	20.93
vgb(A)	Streptogramin	Inhibition of protein synthesis (50S subunit)	Streptogramin B lyase Vgb(A)	21.35
emrC	Efflux	Variable	Multidrug efflux transporter outer membrane subunit EmrC	21.29
ttgB	Efflux	Variable	Multidrug efflux RND transporter permease subunit TtgB	21.19
tbtA	Efflux	Variable	Multidrug efflux RND transporter periplasmic adaptor subunit TbtA	22.08
mtrC	Efflux	Variable	Multidrug efflux RND transporter periplasmic adaptor subunit MtrC	22.08
mexX	Efflux	Variable	Multidrug efflux RND transporter periplasmic adaptor subunit MexX	21.06
smeF	Efflux	Variable	Multidrug efflux RND transporter outer membrane subunit SmeF	21.59
smfY	Efflux	Variable	Multidrug efflux MFS transporter SmfY	19.68
emrA	Efflux	Variable	Multidrug efflux MFS transporter periplas‐mic adaptor subunit EmrA	21.72
mexE	Efflux	Variable	MexE family multidrug efflux RND transporter periplasmic adaptor subunit	21.06
emhC	Efflux	Variable	Efflux RND transporter outer membrane subunit EmhC	21.33
cmlR	Phenicol	Inhibition of protein synthesis (50S subunit)	Chloramphenicol efflux MFS transporter CmlR	21.72
ranA	Aminoglycoside	Inhibition of protein synthesis (30S subunit)	Multidrug efflux ABC transporter ATP‐binding subunit RanA	21.81
bcrA	Bacitracin	Inhibition of cell wall synthesis	Bacitracin resistance ABC transporter ATP‐binding subunit BcrA	21.08
*Downregulated*				
qepA	Quinolone	Inhibition of nucleic acid synthesis (DNA gyrase)	Fluoroquinolone efflux MFS transporter QepA	−21.03
tet(32)	Tetracycline	Inhibition of protein synthesis (30S subunit)	Tetracycline resistance ribosomal protection protein Tet(32)	−20.97
taeA	Pleuromutilin	Inhibition of protein synthesis (50S subunit)	ABC‐F type ribosomal protection protein TaeA	−8.61
vanH‐A	Glycopeptide	Inhibition of cell wall synthesis	D‐lactate dehydrogenase VanH‐A	−24.69

MANOVA on the summed TPM values for each drug class revealed a significant warming × drug class interaction (*p* = 0.001). Lincosamide/Streptogramin was the drug class with the highest Cohen's *D* (1.34), indicating a relatively large magnitude of greater expression in the warmed treatment (Table 3). Nineteen of the 22 detected drug classes had Cohen's *D* values > 1. In this context, we find support for our second hypothesis that the effect of warming varies by drug class.

**TABLE 3:** Cohen's *D* effect sizes for the differences in TPM values between warmed and control treatments for each drug class.

Drug class	Mechanism of action	Mean control (TPM)	Mean warmed (TPM)	Cohen's *D*
Beta‐Lactam	Inhibition of cell wall synthesis	0.81	7.27	1.26
Fosfomycin	Inhibition of cell wall synthesis	0.46	5.43	1.21
Glycopeptide	Inhibition of cell wall synthesis	19.47	124.42	1.14
Efflux	Variable	9.79	45.5	1.15
Phenicol/Quinolone	Variable	0.20	1.42	1.22
Bleomycin	Inhibition of DNA synthesis	0.05	0.48	1.01
Sulfonamide	Inhibition of DNA synthesis	0.62	5.30	1.19
Rifamycin	Inhibition of RNA synthesis	0.29	4.84	1.15
Aminoglycoside	Inhibition of protein synthesis (30S)	0.61	5.98	1.18
Streptothricin	Inhibition of protein synthesis (30S)	0.05	0.41	1.05
Tetracenomycin	Inhibition of protein synthesis (30S)	3.64	16.74	0.97
Tetracycline	Inhibition of protein synthesis (30S)	16.53	152.09	1.15
Tuberactinomycin	Inhibition of protein synthesis (30S)	0.00	0.00	
Lincosamide	Inhibition of protein synthesis (50S)	0.31	0.89	0.64
Lincosamide/Macrolide	Inhibition of protein synthesis (50S)	0.00	0.00	
Lincosamide/Macrolide/Streptogramin	Inhibition of protein synthesis (50S)	0.50	4.12	1.16
Lincosamide/Streptogramin	Inhibition of protein synthesis (50S)	0.01	0.56	1.34
Macrolide	Inhibition of protein synthesis (50S)	0.92	9.65	1.18
Macrolide/Streptogramin	Inhibition of protein synthesis (50S)	0.00	0.00	
Phenicol	Inhibition of protein synthesis (50S)	2.44	18.02	1.18
Streptogramin	Inhibition of protein synthesis (50S)	0.96	4.30	0.97
Thiostrepton	Inhibition of protein synthesis (50S)	0.43	3.48	1.11

#### 3.2.3 Taxonomy

**FIGURE 3:**
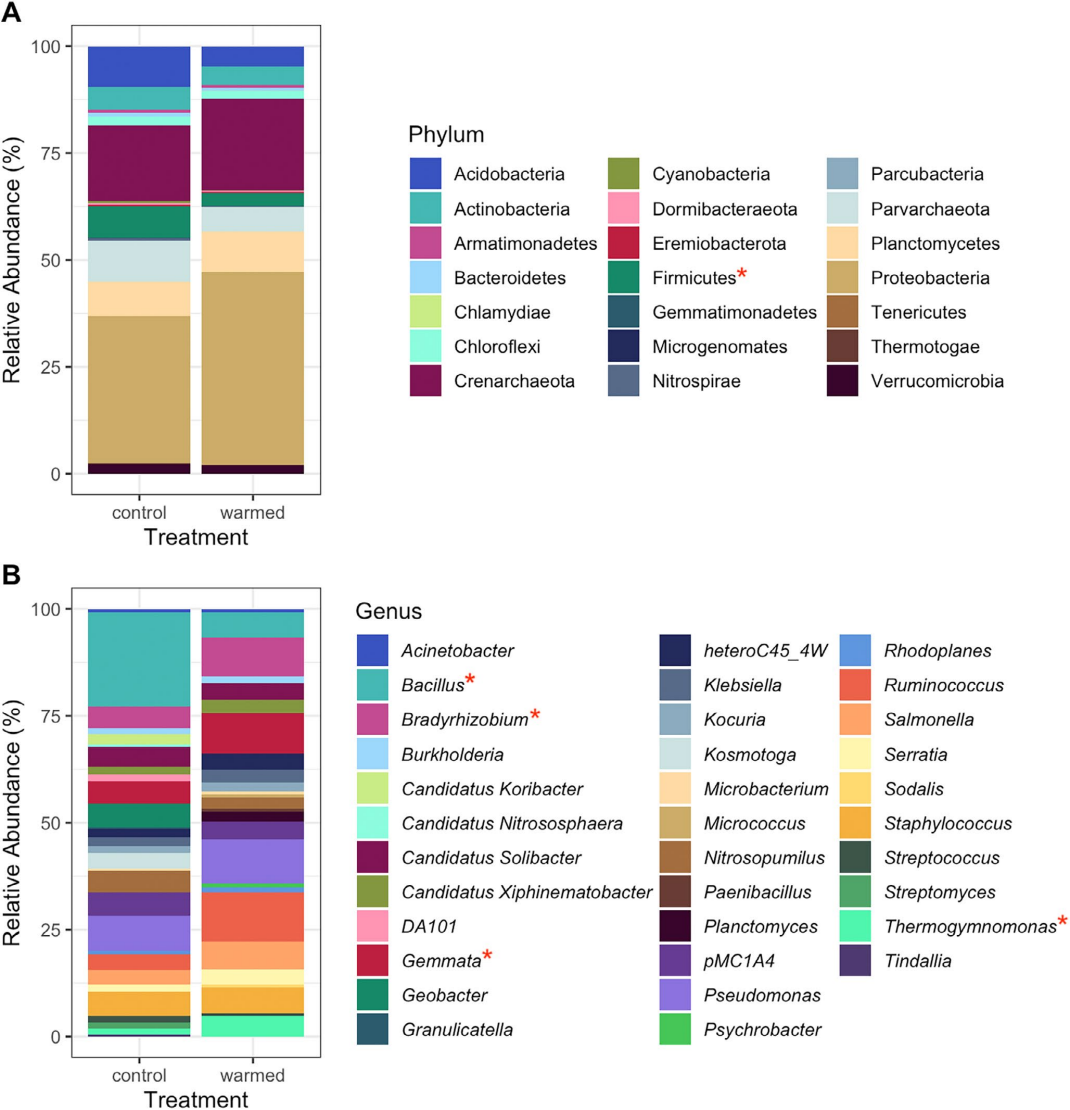
Relative abundances of the most common bacterial phyla (A) and genera (B) of control and warmed communities. Stacked bars are means of four replicates. A red asterisk denotes a significant change in abundance between treatments.

We detected 88 and 86 phyla for the control and warmed communities, respectively (Figure 3A). Transcripts of Proteobacteria were the most abundant phylum across both treatments, followed by Crenarchaeota. For control communities, Acidobacteria was the third most abundant phylum, while Planctomycetes was the third most abundant phylum for warmed communities. In general, phyla did not differ significantly in relative abundance between treatments. However, one exception was the phylum Firmicutes, whose transcript abundance was significantly reduced in the warmed treatment (*p* = 0.012).

The 15 most common genera tended to differ between the control and warmed communities (Figure 3B). The predicted abundance of the genus *Bacillus* was significantly lower in the warmed treatment (*p* = 0.005), while the predicted abundances of the genera *Gemmata*, *Bradyrhizobium* and *Thermogymnomonas* were significantly higher in the warmed treatment (*p* = 0.005, *p* = 0.002 and *p* = 0.005, respectively).

## 4 Discussion

**FIGURE 4:**
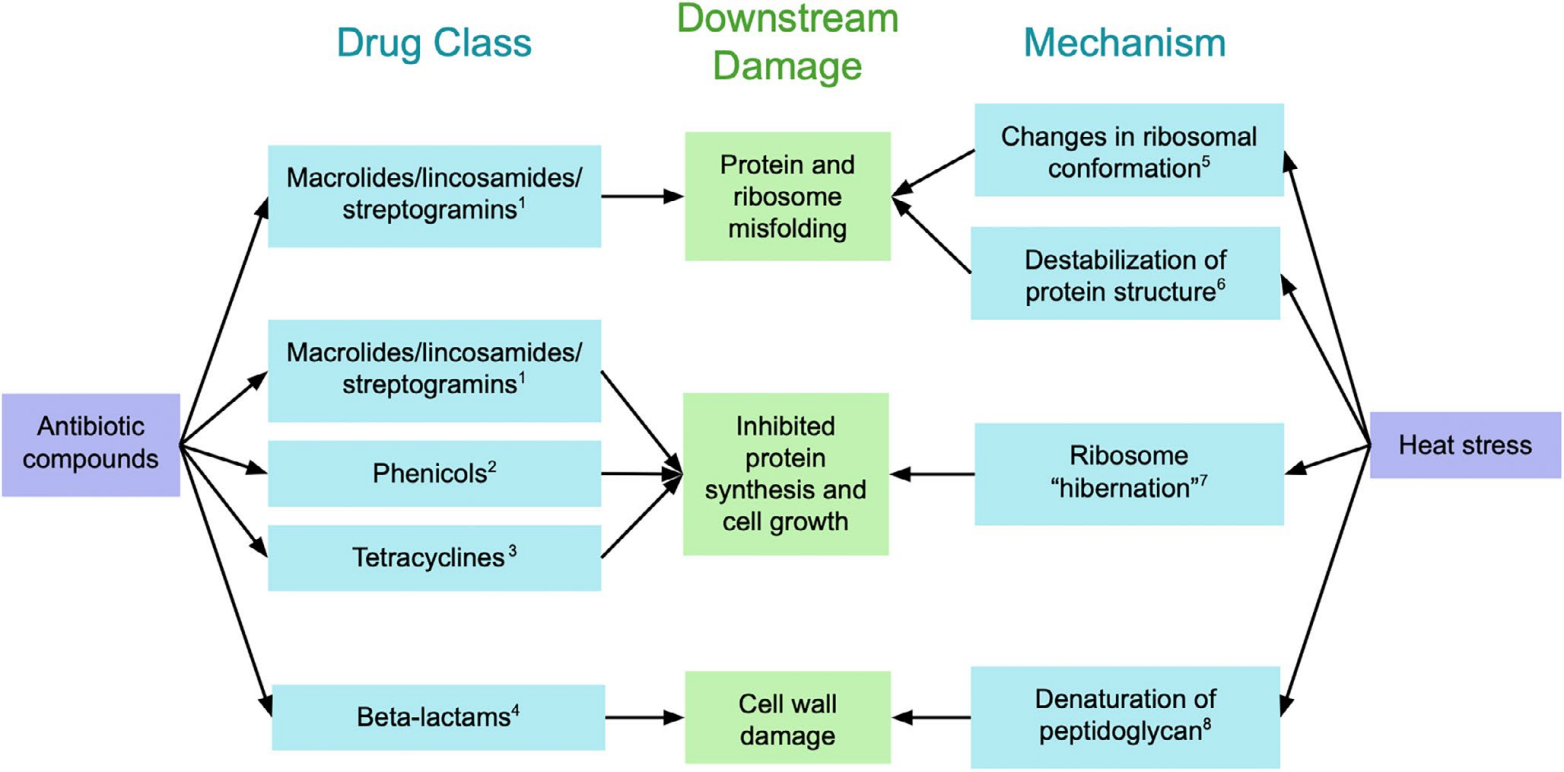
Conceptual figure summarising common downstream damage of heat stress and antibiotic compounds. References: ^1^Edelstein (2004), ^2^Jardetzky (1963), ^3^Chopra and Roberts (2001), ^4^Bush and Bradford (2016), ^5^Al Refaii and Alix (2009), ^6^Anfinsen and Scheraga (1975), ^7^Yoshida and Wada (2014), ^8^Mackey et al. (1991).

A growing body of evidence has indicated that increased temperatures are coupled with greater AMR (Li, Sun, et al. 2022; Li et al. 2023). Understanding how increasing temperatures may exacerbate the spread of AMR in soil can help us assess the risk of human exposure. Our study addressed a knowledge gap regarding the relationship between temperature and AMR within soil bacterial communities. After evaluating AMR gene presence in publicly available soil genomes and AMR gene expression in a warming field experiment, we found an overall positive relationship between temperature and AMR prevalence (Figures 2 and 4). Furthermore, the effect of temperature on AMR transcription and gene presence varied by drug class (Tables 1 and 3). In a previous study with the same sequences from the field site, other TPM measurements, mostly regarding metabolism and enzyme production, were found to be similar between treatments (Romero‐Olivares et al. 2019).

### 4.1 AMR Analysis of Soil Bacterial Genomes (Genome Study)

#### 4.1.1 Higher Reported Optimal Temperature is Associated With a Greater Number of Encoded AMR Genes of Specific Classes in Soil Bacteria

We found a positive relationship between reported optimal growth temperature and the number of encoded AMR genes in soil bacterial genomes (Figure 2). The optimal growth temperature of a bacterium is the temperature at which its growth rate and metabolic activity peak, and it often governs the geographic distribution of bacteria (Ratkowsky et al. 1982; Waksman and Gerretsen 1931). Bacteria and fungi respond to warming by increasing their optimal growth temperatures through adaptation (Bárcenas‐Moreno et al. 2009; Donhauser et al. 2020; Romero‐Olivares et al. 2015; Rousk et al. 2012). Additionally, environmental temperature can be correlated with increased AMR abundance and the rate at which bacteria accumulate resistance over time (Kaba et al. 2020; MacFadden et al. 2018; McGough et al. 2020). Warming is also linked to the dominance of rapidly growing and stress‐resistant taxa in a study conducted in alpine soils (Donhauser et al. 2020). In this context, it is possible that bacteria with higher optimal growth temperatures have accumulated more AMR genes to mitigate heat stress. Specifically, we discovered the strongest positive relationships between reported optimal temperature and the fraction of normalised encoded AMR genes in the drug classes tetracycline, lincosamide/macrolide, lincosamide/macrolide/streptogramin and phenicol. These drug classes share the mechanism of inhibiting protein synthesis.

High temperatures significantly affect bacterial ribosomes and protein synthesis (Starosta et al. 2014; VanBogelen and Neidhardt 1990). First, heat can cause the destabilisation of protein structures, leading to misfolding and aggregation (Anfinsen and Scheraga 1975; Schramm et al. 2020; Yura et al. 1993). High temperatures also destabilise ribosomal RNA (rRNA), causing conformational changes in ribosomal subunit precursors (Al Refaii and Alix 2009; Giudice et al. 2023). Additionally, heat stress can cause ribosomes to ‘hibernate’, reducing total protein synthesis and cell growth (Maki and Yoshida 2021; Wang, Liang, et al. 2020; Yoshida and Wada 2014). Overall, the effects of heat stress share notable commonalities with downstream effects of antibiotics that target protein synthesis.

Tetracyclines are broad spectrum antibiotic compounds targeting gram‐positive and gram‐negative bacteria (Chopra and Roberts 2001). However, decades of the widespread use of tetracyclines have led to the acquisition of tetracycline resistance across numerous bacterial genera (Roberts 2005). Additionally, resistance to tetracyclines is documented in environmental samples (Bryan et al. 2004; Nascimento et al. 2003). Evidence from previous studies supports the possible interaction between tetracycline resistance and temperature, because these stressors act similarly on bacterial ribosomes(Cruz‐Loya et al. 2019; VanBogelen and Neidhardt 1990). Tetracycline inhibits bacterial protein synthesis by binding to the 30S ribosomal subunit (Chopra and Roberts 2001). This mechanism blocks tRNA from binding to the ribosomal A site, preventing the addition of amino acids to a growing polypeptide chain (Roberts and Schwarz 2016). Bacterial growth is inhibited until the drug is removed. Heat can also reduce protein synthesis and bacterial growth by causing an increase in denatured or hibernating ribosomes (Giudice et al. 2023; Yoshida and Wada 2014). In this context, high temperatures and tetracyclines induce similar detrimental effects on bacterial cells. Thus, tetracycline resistance genes may be favoured by bacteria with higher optimal growth temperatures. Our results suggest tetracycline resistance genes are present within soil bacterial genomes and are positively correlated with reported optimal growth temperature.

A growing field of research reports that macrolide/lincosamide/streptogramin resistance genes are found only in environmental bacteria (Bombaywala et al. 2021; Roberts 2011; Toledo et al. 2020). Macrolide, lincosamide and streptogramin (MLS) antibiotics (and resistance genes) are often grouped together due to overlapping binding sites targeting bacterial ribosomes (Edelstein 2004). Specifically, MLS antibiotics target the 50S ribosomal subunit to inhibit protein synthesis (Vannuffel and Cocito 1996). MLS agents constrain the activity of the peptidyl transferase centre of the 50S subunit, preventing peptide elongation and exit (Hansen et al. 1999; Tenson et al. 2003). They can also disrupt the assembly of the 50S subunit, leading to incorrectly folded rRNA (Edelstein 2004; Usary and Champney 2001). In this context, MLS agents and increased temperature can inflict similar damage since increased temperature can also cause conformational changes in rRNA and greatly reduce protein synthesis (Giudice et al. 2023; Wang, Liang, et al. 2020; Yoshida and Wada 2014). Bacteria often carry genes that confer resistance to two or more of these classes (Sutcliffe and Leclercq 2002). Interestingly, numerous MLS resistance genes, a majority encoding an rRNA methylase or efflux pump, have only been detected in environmental bacteria (Nash et al. 2006; Roberts 2011). Furthermore, a previous study of mice faeces suggests environmental heat stress may promote MLS resistant bacteria (Yi et al. 2024). Taken together, our finding of a positive relationship between reported optimal growth temperature and encoded MLS resistance genes in soil bacteria genomes is consistent with these studies.

Phenicol compounds, like MLS compounds, interfere with the 50S ribosomal subunit to inhibit protein synthesis (Jardetzky 1963; Wolfe and Hahn 1965). Specifically, the compound impairs the activity of peptidyl transferase, thereby preventing the formation of peptide bonds (Fisch and Bryskier 2014). Heat can also detrimentally affect peptidyl transferase activity because of increasing structural stability of the ribosome (Njenga et al. 2023). Since both phenicols and increased temperatures inhibit peptidyl transferase activity and subsequent protein synthesis, it is possible that phenicol resistance genes are favoured among bacteria with higher optimal growth temperatures.

Other mechanisms may increase AMR gene presence under warming. Another possible cause is increased mutation and recombination rates (Foster 2005). To survive stressful conditions including temperature stress, bacteria can alter gene expression and mutation rates (Foster 2007). For example, heat shock induced upregulation of GroE increases the activity of error‐prone DNA polymerase IV (Layton and Foster 2005; MacLean et al. 2013). In this context, it is possible that the observed increase in AMR gene presence at higher temperatures resulted from heat shock‐induced mutations. Additionally, the effectiveness of different antibiotic compounds varies at different temperatures (Cruz‐Loya et al. 2021). For example, temperature modulates membrane permeability and rigidity, which in turn can affect the uptake of antibiotic compounds (Loughman et al. 2016; Rahmati‐bahram et al. 1995). Therefore, if an antibiotic compound becomes more effective at increased temperatures, bacteria that encode resistance genes against that antibiotic will be selected for.

### 4.2 AMR Analysis of Warmed Soil Metatranscriptomes (Case Study)

#### 4.2.1 Antimicrobial Gene Upregulation Across Several Drug Classes Was Associated With Warming

For the case study conducted in a remote Alaskan boreal forest, we found a large positive Cohen's *D* effect size for several drug classes regarding the effect of warming on TPM measurements. The drug class with the largest effect size was lincosamide/streptogramin (Table 3). These drug classes are often grouped together due to having overlapping binding sites (Edelstein 2004). Lincosamide resistance is mainly linked to efflux pumps, rRNA methylation, or adenylation of the lincosamide compound itself (Morar et al. 2009; Nolivos et al. 2019; Spížek and Řezanka 2017; Yang et al. 2024). Similarly, mechanisms conferring resistance to streptogramins include efflux, ribosomal modification and antibiotic modification (Johnston et al. 2005). Furthermore, lincosamide‐ and streptogramin‐targeting AMR genes are documented often in the environment (Bombaywala et al. 2021; Roberts 2005, 2011; Yang et al. 2024).

Beta‐lactam TPM measurements also had a large Cohen's *D* effect size. Beta‐lactam compounds bind to proteins involved in peptidoglycan cross‐linking, thereby disrupting cell wall formation (Bush and Bradford 2016). Similarly, heat stress can also interfere with and denature the peptidoglycan cell wall (Ebrahimi et al. 2018; Mackey et al. 1991; Mueller and Levin 2020; Tonyali et al. 2019). Given their similar downstream effects, it is possible that beta‐lactam genes are transcribed at increased temperatures. Beta‐lactam resistant bacteria have been documented in environmental samples (Eltzov et al. 2012; Levy and Bonnie 2004; Schages et al. 2020). Another soil study found that climate warming increased proportions of certain AMR genes including beta‐lactams (Li, Sun, et al. 2022). In this context, our result is consistent with previous literature.

Finally, phenicol/quinolone antibiotics have been used since the 1950s and 1960s as broad‐spectrum antibiotics (Pham et al. 2019; Roberts and Schwarz 2016). Phenicol compounds prevent protein synthesis by binding to the 50S ribosome (Schlünzen et al. 2001). Quinolone antibiotics target DNA gyrase and topoisomerase IV, thereby inhibiting DNA synthesis (Anderson and Osheroff 2005; Pham et al. 2019). Phenicol and quinolone resistance genes within plasmids are readily transferred between bacteria in the environment (Hooper and Jacoby 2015; Roberts and Schwarz 2016).

There may be other causes of increased AMR gene transcription under warming other than common downstream damage. The production of antibiotic compounds by microbes is temperature dependent, which could lead to increases or decreases in subsequent AMR gene transcription (James and Edwards 1989; Ohno et al. 1995). For example, microbes may reduce transcription of antibiotic compounds at temperatures where the compound is less effective (Raaijmakers et al. 2002). High temperature also increases metabolic rate (Clarke and Fraser 2004; Ratkowsky et al. 1982). In this case, bacteria may increase transcription of antibiotic compounds as a defence mechanism if they have more expendable energy (Martínez and Rojo 2011).

This study was conducted in a relatively remote field experiment, so it is likely that any antimicrobial compounds in the soil were produced by bacteria as opposed to being introduced by anthropogenic interference. Bacteria often produce antimicrobials as a competitive mechanism to eliminate others (Gottlieb 1976). Although there are a several well‐studied cases of the natural production of antibiotic compounds by specific genera, more genera and genes may yet be discovered (Mullis et al. 2019; Tiwari and Gupta 2012). Previous DNA‐based studies in natural systems, though scarce, have also found warming effects on the enrichment of AMR and stress‐resistant genes (Donhauser et al. 2020; Li, Sun, et al. 2022). Nevertheless, we are unaware of any previously published studies that examine transcripts of AMR genes under warming in the field.

It is also possible that decreased soil moisture owing to increased evapotranspiration in experimental plots led to increases in AMR gene transcription. Lower moisture levels cause desiccation stress, leading to a stress response that shares common features with AMR mechanisms (Abdelhamid and Yousef 2020; Al‐Nabulsi et al. 2011; Farrow et al. 2018; Gayoso et al. 2014). For example, desiccation and oxidative stress stemming from low moisture disrupt cell membrane integrity, DNA replication and protein folding (Csonka 1989; García 2011; Łupkowska et al. 2023). These stresses also lead to biofilm formation and increased efflux of toxic molecules and ions out of the cell (Csonka and Hanson 1991; McKew et al. 2011; Potts 1994). Indeed, a growing body of research suggests that AMR could be intensified through multiple stressor interaction networks including heavy metal exposure, UV radiation, pH and repeated freezing and thawing in addition to drought stress (Cruz‐Loya et al. 2019; Farrow et al. 2018; Kong et al. 2024; Li et al. 2021; Nguyen et al. 2019; Seiler and Berendonk 2012; Venglarcik et al. 1983). Since these stressors can cause similar downstream damage to bacterial cells, overlapping defence mechanisms that confer protection from multiple stressors are selected for (Battesti et al. 2011; Dragosits et al. 2013; Somorin et al. 2016; Święciło 2016). Moreover, synergistic effects of simultaneous stressors can intensify AMR beyond levels induced by a single stressor (Rodríguez‐Verdugo et al. 2020). Overall, the manifold interactions between stressors in the field underscore the importance of measuring and considering these factors to better understand and manage environmental AMR.

#### 4.2.2 Upregulated Heat Resistance Mechanisms Were Present in Warmed Communities

We found elevated levels of heat resistance associated transcripts sHsp20 (small heat shock protein 20), sHsp20‐GI, KefB‐GI and Trx‐GI (Table S1). SHsp20 and sHsp20‐GI are molecular chaperones that prevent the aggregation of misfolded proteins, conferring heat and oxidative stress resistance (Haslbeck et al. 2005; Kamal et al. 2021; Kappé et al. 2002). KefB‐GI is an H^+^/K^+^ antiporter which helps cells maintain a stable pH, mitigating oxidative stress (MacLean et al. 1998; Wang, Fang, et al. 2020). Finally, Trx‐GI is a gene‐induced (GI) thioredoxin, a protein contributing to redox homeostasis, which is an important mechanism against oxidative stress (Carmel‐Harel and Storz 2000). Interestingly, these heat resistance genes are clustered together in a transferable ‘genomic island’ known as the transmissible locus of stress tolerance (tLST, previously termed the locus of heat resistance or LHR) (Kamal et al. 2021). This gene cluster contains several genes that confer heat, oxidative stress and antibiotic resistance and it is easily transferred among Proteobacteria (Kamal et al. 2021). Many studies suggest anthropogenic activities and increased temperature create a selective pressure that favours microbes possessing the tLST island (Boll et al. 2017; Marti et al. 2016; Zhi et al. 2016). Additionally, our taxonomic data corroborates the presence of these heat resistance genes. Genera documented as possessing this cluster of stress resistance genes include *Salmonella*, *Burkholderia* and *Klebsiella*, which were detected at 4.9%, 1.7% and 2.0% abundance on average in the warmed plots, respectively (Kamal et al. 2021).

#### 4.2.3 Taxonomical Changes Were Observed in Warmed Communities

*Gemmata*, one of the genera significantly more abundant in the warmed treatment (Figure 3B), includes species with a wide range of resistance mechanisms (Aghnatios and Drancourt 2016). Specifically, *Gemmata* possess genes that provide multi‐drug resistance, including multi‐drug resistant efflux pumps and genes conferring resistance to fluoroquinolones (Ivanova et al. 2021). *Gemmata* species are well documented in environmental soil, aquatic ecosystems and wastewater (Buckley et al. 2006; Chouari et al. 2003; Griepenburg et al. 1999; Santarella‐Mellwig et al. 2013; Seiler and Berendonk 2012). *Gemmata* has also been detected in human faeces, blood and on skin (Cayrou et al. 2013; Drancourt et al. 2014; Kong et al. 2012).

The genus *Bradyrhizobium* was also significantly more abundant in warmed plots (Figure 3B). Certain species of *Bradyrhizobium* have demonstrated resistance to several antibiotics, including tetracycline and vancomycin, drug classes for which we found resistance genes in our data (Kuykendall et al. 1988; Mueller et al. 1988; Saeki et al. 2012). *Thermogymnomonas*, significantly more abundant in warmed plots, though not extensively studied, is notable for thriving in higher temperatures (Itoh et al. 2007).

Overall, changes in relative abundance of taxa tended to coincide with changes in differential expression of AMR genes. Shifts in temperature frequently alter microbial community composition (Glassman et al. 2018). Although much community composition research is dedicated to determining the consequences of warming on ecosystem function and microbial productivity (Glassman et al. 2018; Romero‐Olivares et al. 2019), there is growing attention on the influence of temperature on the distribution and activity of infectious microbes (Gorris et al. 2019; Ladau et al. 2018). Our results are consistent with other studies that found greater abundances of stress‐tolerant or AMR resistant taxa at warmer temperatures (Allison and Treseder 2008; MacFadden et al. 2018; Pärnänen et al. 2019). Uncovering the relationship between warming and AMR allows us to anticipate and mitigate the possible consequences on human health and natural ecosystems. Increased prevalence of stress‐tolerant or resistant taxa within microbial communities can increase the reservoir of resistance (Grenni et al. 2018; Rodríguez‐Verdugo et al. 2020).

It is challenging to capture the complexity of soil microbial communities using a solely metatranscriptomic approach, mostly owing to the fragility of RNA. While we acknowledge that using RNA alone might produce different results than both RNA and DNA (Bashiardes et al. 2016), our primary interest was to compare AMR abundance between the warmed and control treatment. Since both treatment groups were subjected to identical methods of data collection and analysis, any issues related to RNA fragility or fragmentation are likely to be consistent across treatments. Given the dynamic nature of gene expression, future studies that account for temporal variation in gene expression through repeated sampling while also measuring antibiotic compounds present in the soil will be valuable. Care is also warranted in interpreting the genome analysis, as we used metadata values provided by users of the BV‐BRC database. These metadata may vary in accuracy or consistency. Additionally, our discussion primarily focused on results from normalised values of AMR gene presence. Although normalised and absolute values generally coincided, we acknowledge that normalised values may obscure changes in genome size or the absolute abundance of AMR genes.

Overall, warming was associated with more antimicrobial resistance in this relatively undisturbed natural ecosystem. Our results emphasise the potential consequences of global warming on the reservoir of resistance. It is well documented that global change can alter the circulation and occurrence of infectious diseases (Boxall et al. 2009; Epstein 2001; MacFadden et al. 2018). If warming is upregulating or selecting for AMR, these genes could propagate among bacterial communities. This response could result in an increased reservoir of resistance genes, facilitating the acquisition of resistance by pathogenic bacteria (Rodríguez‐Verdugo et al. 2020). What begins as a physiological response to warming could induce changes in bacterial gene expression that may exacerbate the global spread of resistance.

## 5 Conclusion

In summary, warming may promote AMR in soil bacterial communities. This study includes one of the first experiments evaluating how AMR expression is regulated by temperature in a field setting. As global temperatures rise, bacteria may accumulate or upregulate expression of AMR genes to better tolerate environmental stress. If this trend is widespread, it may exacerbate the spread of resistant bacteria, increasing the risk of human, plant and animal exposure. With the abundance of publicly available genome and transcriptome data, it is possible to gain quick insight into the soil resistome in other field studies, which will elucidate the scale and magnitude of resistance genes in soil. This approach could lead to new ways to mitigate the antimicrobial crisis.

## Author Contributions

**Melanie T. Hacopian:** writing – original draft, writing – review and editing, visualization, methodology, investigation, formal analysis, conceptualization, software. **Alberto Barrón‐Sandoval:** writing – review and editing, methodology. **Adriana L. Romero‐Olivares:** data curation, methodology, writing – review and editing, resources, funding acquisition. **Renaud Berlemont:** data curation, software, methodology, writing – review and editing. **Kathleen K. Treseder:** methodology, resources, supervision, funding acquisition, investigation, conceptualization, writing – review and editing.

## Conflicts of Interest

The authors declare no conflicts of interest.

## Supporting information


**Figure S1.** Positive relationship between reported optimal growth temperature and (A) the number of encoded AMR genes normalised by the number of protein‐encoding genes (PEGs) or (B) the number of total encoded AMR genes. Each symbol represents a bacterial genome isolated from soil (*n* = 253 genomes). Colour represents phylum. Isolates with an optimal temperature of 37°C were removed.
**Figure S2.** Differential expression of AMR (A) and heat resistance (B) transcripts in soil microbial communities undergoing temperature warming. The *x*‐axis represents log2 fold change of transcript counts in warming plots compared with controls. Plots show the 112,226 transcripts with > 50 counts between all plots. Each symbol represents one transcript. Symbols in the green region are significantly upregulated in the warmed treatment, while symbols in the blue region are significantly downregulated. Filled symbols are transcripts associated with AMR (A) and heat resistance (B). Open symbols are other transcripts. Dotted lines represent significance cut‐offs for the log10 adjusted *p* value and log2 fold change.

**Table S1.** Significantly upregulated and downregulated heat resistance transcripts with average fold change values (*p* < 0.05 for all).

## Data Availability

Sequences used for the case study are available for download on JGI's data portal under the following project IDs: 1107‐496, 1107‐499, 1107‐504, 1107‐507, 1107‐509, 1107‐514, 1107‐519 and 1107‐520. Genome identifiers for the genomes included in the bioinformatic investigation and code used for the study are deposited at https://github.com/melaniehacopian/AMR_workflow. Other data will be made available upon request.

## References

[emi70097-bib-0001] Abdelhamid, A. G. , and A. E. Yousef . 2020. “Collateral Adaptive Responses Induced by Desiccation Stress in *Salmonella enterica* .” LWT 133: 110089. 10.1016/J.LWT.2020.110089.

[emi70097-bib-0002] Aghnatios, R. , and M. Drancourt . 2016. “Gemmata Species: Planctomycetes of Medical Interest.” Future Microbiology 11, no. 5: 659–667. 10.2217/FMB-2015-0001/FORMAT/EPUB. 27158864

[emi70097-bib-0003] Al Refaii, A. , and J. H. Alix . 2009. “Ribosome Biogenesis is Temperature‐Dependent and Delayed in *Escherichia coli* Lacking the Chaperones DnaK or DnaJ.” Molecular Microbiology 71, no. 3: 748–762. 10.1111/J.1365-2958.2008.06561.X. 19054328

[emi70097-bib-0004] Allison, S. D. , and K. K. Treseder . 2008. “Warming and Drying Suppress Microbial Activity and Carbon Cycling in Boreal Forest Soils.” Global Change Biology 14, no. 12: 2898–2909. 10.1111/J.1365-2486.2008.01716.X.

[emi70097-bib-0005] Al‐Nabulsi, A. A. , T. M. Osaili , N. A. Z. Elabedeen , et al. 2011. “Impact of Environmental Stress Desiccation, Acidity, Alkalinity, Heat or Cold on Antibiotic Susceptibility of *Cronobacter sakazakii* .” International Journal of Food Microbiology 146, no. 2: 137–143. 10.1016/J.IJFOODMICRO.2011.02.013. 21402424

[emi70097-bib-0006] Anderson, V. , and N. Osheroff . 2005. “Type II Topoisomerases as Targets for Quinolone Antibacterials Turning Dr. Jekyll Into Mr. Hyde.” Current Pharmaceutical Design 7, no. 5: 337–353. 10.2174/1381612013398013. 11254893

[emi70097-bib-0007] Andrews, S. 2010. “FastQC: A Quality Control Tool for High Throughput Sequence Data.” [Online]. Babraham Bioinformatics. http://www.bioinformatics.babraham.ac.uk/projects/fastqc/.

[emi70097-bib-0008] Anfinsen, C. B. , and H. A. Scheraga . 1975. “Experimental and Theoretical Aspects of Protein Folding.” Advances in Protein Chemistry 29: 205–300. 10.1016/S0065-3233(08)60413-1. 237413

[emi70097-bib-0009] Bandyopadhyay, S. , and I. Samanta . 2020. “Antimicrobial Resistance in Agri‐Food Chain and Companion Animals as a Re‐Emerging Menace in Post‐COVID Epoch: Low‐ and Middle‐Income Countries Perspective and Mitigation Strategies.” Frontiers in Veterinary Science 7, no. 620: 1–19. 10.3389/fvets.2020.00620. 33195500 PMC7581709

[emi70097-bib-0010] Bárcenas‐Moreno, G. , M. G. Brandón , J. Rousk , and E. Bååth . 2009. “Adaptation of Soil Microbial Communities to Temperature: Comparison of Fungi and Bacteria in a Laboratory Experiment.” Global Change Biology 15, no. 12: 2950–2957. 10.1111/J.1365-2486.2009.01882.X.

[emi70097-bib-0011] Bashiardes, S. , G. Zilberman‐Schapira , and E. Elinav . 2016. “Use of Metatranscriptomics in Microbiome Research.” Bioinformatics and Biology Insights 10: 19–25. 10.4137/BBI.S34610. 27127406 PMC4839964

[emi70097-bib-0012] Battesti, A. , N. Majdalani , and S. Gottesman . 2011. “The RpoS‐Mediated General Stress Response in *Escherichia coli* .” Annual Review of Microbiology 65: 189–213. 10.1146/ANNUREV-MICRO-090110-102946. PMC7356644 21639793

[emi70097-bib-0013] Bauer, D. F. 1972. “Constructing Confidence Sets Using Rank Statistics.” Journal of the American Statistical Association 67, no. 339: 687. 10.2307/2284469.

[emi70097-bib-0014] Baumgardner, D. J. 2012. “Soil‐Related Bacterial and Fungal Infections.” Journal of the American Board of Family Medicine 25, no. 5: 734–744. 10.3122/JABFM.2012.05.110226. 22956709

[emi70097-bib-0015] Bolger, A. M. , M. Lohse , and B. Usadel . 2014. “Trimmomatic: A Flexible Trimmer for Illumina Sequence Data.” Bioinformatics 30, no. 15: 2114–2120. 10.1093/BIOINFORMATICS/BTU170. 24695404 PMC4103590

[emi70097-bib-0016] Boll, E. J. , R. Marti , H. Hasman , et al. 2017. “Turn Up the Heat‐Food and Clinical *Escherichia coli* Isolates Feature Two Transferrable Loci of Heat Resistance.” Frontiers in Microbiology 8: 579. 10.3389/FMICB.2017.00579/BIBTEX. 28439262 PMC5383660

[emi70097-bib-0017] Bombaywala, S. , A. Mandpe , S. Paliya , and S. Kumar . 2021. “Antibiotic Resistance in the Environment: A Critical Insight on Its Occurrence, Fate, and Eco‐Toxicity.” Environmental Science and Pollution Research 28, no. 20: 24889–24916. 10.1007/S11356-021-13143-X. 33765260

[emi70097-bib-0018] Boxall, A. B. A. , A. Hardy , S. Beulke , et al. 2009. “Impacts of Climate Change on Indirect Human Exposure to Pathogens and Chemicals From Agriculture.” Environmental Health Perspectives 117, no. 4: 508–514. 10.1289/EHP.0800084. 19440487 PMC2679592

[emi70097-bib-0019] Brauner, A. , O. Fridman , O. Gefen , and N. Q. Balaban . 2016. “Distinguishing Between Resistance, Tolerance and Persistence to Antibiotic Treatment.” Nature Reviews Microbiology 14, no. 5: 320–330. 10.1038/nrmicro.2016.34. 27080241

[emi70097-bib-0020] Brock, T. D. 1978. “The Habitats.” In Springer Series in Microbiology, 12–38. Springer. 10.1007/978-1-4612-6284-8.

[emi70097-bib-0021] Bryan, A. , N. Shapir , and M. J. Sadowsky . 2004. “Frequency and Distribution of Tetracycline Resistance Genes in Genetically Diverse, Nonselected, and Nonclinical *Escherichia coli* Strains Isolated From Diverse Human and Animal Sources.” Applied and Environmental Microbiology 70, no. 4: 2503. 10.1128/AEM.70.4.2503-2507.2004. 15066850 PMC383146

[emi70097-bib-0022] Buckley, D. H. , V. Huangyutitham , T. A. Nelson , A. Rumberger , and J. E. Thies . 2006. “Diversity of Planctomycetes in Soil in Relation to Soil History and Environmental Heterogeneity.” Applied and Environmental Microbiology 72, no. 7: 4522–4531. 10.1128/AEM.00149-06. 16820439 PMC1489350

[emi70097-bib-0023] Burmølle, M. , T. R. Thomsen , M. Fazli , et al. 2010. “Biofilms in Chronic Infections – A Matter of Opportunity – Monospecies Biofilms in Multispecies Infections.” FEMS Immunology and Medical Microbiology 59, no. 3: 324–336. 10.1111/J.1574-695X.2010.00714.X. 20602635

[emi70097-bib-0024] Bush, K. , and P. A. Bradford . 2016. “β‐Lactams and β‐Lactamase Inhibitors: An Overview.” Cold Spring Harbor Perspectives in Medicine 6, no. 8: 1–22. 10.1101/CSHPERSPECT.A025247. PMC4968164 27329032

[emi70097-bib-0025] Cardoso, K. , R. F. Gandra , E. S. Wisniewski , et al. 2010. “DnaK and GroEL Are Induced in Response to Antibiotic and Heat Shock in *Acinetobacter baumannii* .” Journal of Medical Microbiology 59, no. 9: 1061–1068. 10.1099/JMM.0.020339-0. 20576751

[emi70097-bib-0026] Carmel‐Harel, O. , and G. Storz . 2000. “Roles of the Glutathione‐ and Thioredoxin‐Dependent Reduction Systems in the *Escherichia coli* and *Saccharomyces cerevisiae* Responses to Oxidative Stress.” Annual Review of Microbiology 54: 439–461. 10.1146/ANNUREV.MICRO.54.1.439. 11018134

[emi70097-bib-0027] Cayan, D. R. , T. Das , D. W. Pierce , T. P. Barnett , M. Tyree , and A. Gershunova . 2010. “Future Dryness in the Southwest US and the Hydrology of the Early 21st Century Drought.” Proceedings of the National Academy of Sciences of the United States of America 107, no. 50: 21271–21276. 10.1073/pnas.0912391107. 21149687 PMC3003012

[emi70097-bib-0028] Cayrou, C. , B. Sambe , F. Armougom , D. Raoult , and M. Drancourt . 2013. “Molecular Diversity of the Planctomycetes in the Human Gut Microbiota in France and Senegal.” APMIS 121, no. 11: 1082–1090. 10.1111/APM.12087. 23594317

[emi70097-bib-0029] Chopra, I. , and M. Roberts . 2001. “Tetracycline Antibiotics: Mode of Action, Applications, Molecular Biology, and Epidemiology of Bacterial Resistance.” Microbiology and Molecular Biology Reviews 65, no. 2: 232. 10.1128/MMBR.65.2.232-260.2001. 11381101 PMC99026

[emi70097-bib-0030] Chouari, R. , D. Le Paslier , P. Daegelen , P. Ginestet , J. Weissenbach , and A. Sghir . 2003. “Molecular Evidence for Novel Planctomycete Diversity in a Municipal Wastewater Treatment Plant.” Applied and Environmental Microbiology 69, no. 12: 7354. 10.1128/AEM.69.12.7354-7363.2003. 14660385 PMC309898

[emi70097-bib-0031] Clarke, A. , and K. P. P. Fraser . 2004. “Why Does Metabolism Scale With Temperature?” JSTOR 18: 243–251.

[emi70097-bib-0032] Cohen, J. 1988. Statistical Power Analysis for the Behavioral Sciences. Routledge Academic. 10.4324/9780203771587.

[emi70097-bib-0033] Cook, B. I. , T. R. Ault , and J. E. Smerdon . 2015. “Unprecedented 21st Century Drought Risk in the American Southwest and Central Plains.” Science Advances 1, no. 1: e1400082. 10.1126/sciadv.1400082. 26601131 PMC4644081

[emi70097-bib-0034] Cruz‐Loya, M. , T. M. Kang , N. A. Lozano , et al. 2019. “Stressor Interaction Networks Suggest Antibiotic Resistance Co‐Opted From Stress Responses to Temperature.” ISME Journal 13, no. 1: 12–23. 10.1038/s41396-018-0241-7. 30171253 PMC6298959

[emi70097-bib-0035] Cruz‐Loya, M. , E. Tekin , T. M. Kang , et al. 2021. “Antibiotics Shift the Temperature Response Curve of *Escherichia coli* Growth.” MSystems 6, no. 4: 1–15. 10.1128/MSYSTEMS.00228-21/SUPPL_FILE/MSYSTEMS.00228-21-SF002.EPS. PMC8422994 34282938

[emi70097-bib-0036] Csonka, L. N. 1989. “Physiological and Genetic Responses of Bacteria to Osmotic Stress.” Microbiological Reviews 53, no. 1: 121. 10.1128/MR.53.1.121-147.1989. 2651863 PMC372720

[emi70097-bib-0037] Csonka, L. N. , and A. D. Hanson . 1991. “Prokaryotic Osmoregulation: Genetics and Physiology.” Annual Review of Microbiology 45: 569–606. 10.1146/ANNUREV.MI.45.100191.003033. 1741624

[emi70097-bib-0038] Dcosta, V. M. , C. E. King , L. Kalan , et al. 2011. “Antibiotic Resistance is Ancient.” Nature 477, no. 7365: 457–461. 10.1038/nature10388. 21881561

[emi70097-bib-0039] DeSantis, T. Z. , P. Hugenholtz , N. Larsen , et al. 2006. “Greengenes, a Chimera‐Checked 16S rRNA Gene Database and Workbench Compatible With ARB.” Applied and Environmental Microbiology 72, no. 7: 5069–5072. 10.1128/AEM.03006-05. 16820507 PMC1489311

[emi70097-bib-0040] Dixon, P. 2003. “VEGAN, a Package of R Functions for Community Ecology.” Journal of Vegetation Science 14, no. 6: 927–930. 10.1111/J.1654-1103.2003.TB02228.X.

[emi70097-bib-0041] Do, T. T. , C. Smyth , F. Crispie , C. Burgess , F. Brennan , and F. Walsh . 2023. “Comparison of Soil and Grass Microbiomes and Resistomes Reveals Grass as a Greater Antimicrobial Resistance Reservoir Than Soil.” Science of the Total Environment 857: 159179. 10.1016/J.SCITOTENV.2022.159179. 36191722

[emi70097-bib-0042] Donhauser, J. , P. A. Niklaus , J. Rousk , C. Larose , and B. Frey . 2020. “Temperatures Beyond the Community Optimum Promote the Dominance of Heat‐Adapted, Fast Growing and Stress Resistant Bacteria in Alpine Soils.” Soil Biology and Biochemistry 148: 107873. 10.1016/J.SOILBIO.2020.107873.

[emi70097-bib-0043] Dragosits, M. , V. Mozhayskiy , S. Quinones‐Soto , J. Park , and I. Tagkopoulos . 2013. “Evolutionary Potential, Cross‐Stress Behavior and the Genetic Basis of Acquired Stress Resistance in *Escherichia coli* .” Molecular Systems Biology 9: 1–13. 10.1038/MSB.2012.76. PMC3588905 23385483

[emi70097-bib-0044] Drancourt, M. , T. Prebet , R. Aghnatios , et al. 2014. “Planctomycetes DNA in Febrile Aplastic Patients With Leukemia, Rash, Diarrhea, and Micronodular Pneumonia.” Journal of Clinical Microbiology 52, no. 9: 3453–3455. 10.1128/JCM.01207-14. 24920769 PMC4313204

[emi70097-bib-0045] Ebrahimi, A. , L. N. Csonka , and M. A. Alam . 2018. “Analyzing Thermal Stability of Cell Membrane of Salmonella Using Time‐Multiplexed Impedance Sensing.” Biophysical Journal 114, no. 3: 609. 10.1016/J.BPJ.2017.10.032. 29414707 PMC5985002

[emi70097-bib-0046] Edelstein, P. H. 2004. “Pneumococcal Resistance to Macrolides, Lincosamides, Ketolides, and Streptogramin B Agents: Molecular Mechanisms and Resistance Phenotypes.” Clinical Infectious Diseases 38: 322–329. 10.1086/382687. 15127365

[emi70097-bib-0047] Eltzov, E. , S. Pennybaker , M. Shanit‐Orland , R. S. Marks , and A. Kushmaro . 2012. “Multi‐Resistance as a Tool for Detecting Novel Beta‐Lactam Antibiotics in the Environment.” Sensors and Actuators B: Chemical 174: 342–348. 10.1016/J.SNB.2012.07.059.

[emi70097-bib-0048] Epstein, P. R. 2001. “Climate Change and Emerging Infectious Diseases.” Microbes and Infection 3, no. 9: 747–754. 10.1016/S1286-4579(01)01429-0. 11489423

[emi70097-bib-0049] Farrow, J. M. , G. Wells , and E. C. Pesci . 2018. “Desiccation Tolerance in *Acinetobacter baumannii* is Mediated by the Two‐Component Response Regulator BfmR.” PLoS One 13, no. 10: e0205638. 10.1371/JOURNAL.PONE.0205638. 30308034 PMC6181384

[emi70097-bib-0050] Feldgarden, M. , V. Brover , N. Gonzalez‐Escalona , et al. 2021. “AMRFinderPlus and the Reference Gene Catalog Facilitate Examination of the Genomic Links Among Antimicrobial Resistance, Stress Response, and Virulence.” Scientific Reports 11, no. 12728: 1–9. 10.1038/s41598-021-91456-0. 34135355 PMC8208984

[emi70097-bib-0051] Fisch, A. , and A. Bryskier . 2014. “Phenicols.” Antimicrobial Agents 43: 925–929. 10.1128/9781555815929.CH33.

[emi70097-bib-0052] Foster, P. L. 2005. “Stress Responses and Genetic Variation in Bacteria.” Mutation Research, Fundamental and Molecular Mechanisms of Mutagenesis 569, no. 1–2: 3–11. 10.1016/J.MRFMMM.2004.07.017. 15603749 PMC2729700

[emi70097-bib-0053] Foster, P. L. 2007. “Stress‐Induced Mutagenesis in Bacteria.” Critical Reviews in Biochemistry and Molecular Biology 42, no. 5: 373–397. 10.1080/10409230701648494. 17917873 PMC2747772

[emi70097-bib-0054] Fuchsman, C. A. , R. E. Collins , G. Rocap , and W. J. Brazelton . 2017. “Effect of the Environment on Horizontal Gene Transfer Between Bacteria and Archaea.” PeerJ 2017, no. 9: e3865. 10.7717/PEERJ.3865/SUPP-3. PMC5624296 28975058

[emi70097-bib-0055] García, A. H. 2011. “Anhydrobiosis in Bacteria: From Physiology to Applications.” Journal of Biosciences 36, no. 5: 939–950. 10.1007/S12038-011-9107-0. 22116292

[emi70097-bib-0056] Gayoso, C. M. , J. Mateos , J. A. Méndez , et al. 2014. “Molecular Mechanisms Involved in the Response to Desiccation Stress and Persistence in *Acinetobacter baumannii* .” Journal of Proteome Research 13, no. 2: 460–476. 10.1021/PR400603F/SUPPL_FILE/PR400603F_SI_001.PDF. 24299215

[emi70097-bib-0057] Giudice, E. , S. Georgeault , R. Lavigne , et al. 2023. “Purification and Characterization of Authentic 30S Ribosomal Precursors Induced by Heat Shock.” International Journal of Molecular Sciences 24, no. 4: 3491. 10.3390/IJMS24043491/S1. 36834906 PMC9959188

[emi70097-bib-0058] Glassman, S. I. , C. Weihe , J. Li , et al. 2018. “Decomposition Responses to Climate Depend on Microbial Community Composition.” Proceedings of the National Academy of Sciences of the United States of America 115, no. 47: 11994–11999. 10.1073/PNAS.1811269115/SUPPL_FILE/PNAS.1811269115.SAPP.PDF. 30397146 PMC6255157

[emi70097-bib-0059] Gorris, M. E. , K. K. Treseder , C. S. Zender , and J. T. Randerson . 2019. “Expansion of Coccidioidomycosis Endemic Regions in the United States in Response to Climate Change.” GeoHealth 3, no. 10: 308–327. 10.1029/2019GH000209. 32159021 PMC7007157

[emi70097-bib-0060] Gottlieb, D. 1976. “The Production and Role of Antibiotics in Soil.” Journal of Antibiotics 29, no. 10: 987–1000. 10.7164/ANTIBIOTICS.29.987. 994333

[emi70097-bib-0061] Gottlieb, D. , and P. Shaw . 2013. “Antibiotics: Volume I Mechanism of Action.” https://books.google.com/books?hl=en&lr=&id=0M3yCAAAQBAJ&oi=fnd&pg=PR11&dq=antibiotics+mechanisms+of+action&ots=e1BOFwDxTR&sig=jcas7QINhKJniPfK1oDmira1exQ.

[emi70097-bib-0062] Grenni, P. , V. Ancona , and A. Barra Caracciolo . 2018. “Ecological Effects of Antibiotics on Natural Ecosystems: A Review.” Microchemical Journal 136: 25–39. 10.1016/J.MICROC.2017.02.006.

[emi70097-bib-0063] Griepenburg, U. , N. Ward‐Rainey , T. S. Mohamed , et al. 1999. “Phylogenetic Diversity, Polyamine Pattern and DNA Base Composition of Members of the Order Planctomycetales.” International Journal of Systematic Bacteriology 49: 689–696. 10.1099/00207713-49-2-689. 10319492

[emi70097-bib-0064] Hansen, L. H. , P. Mauvais , and S. Douthwaite . 1999. “The Macrolide‐Ketolide Antibiotic Binding Site is Formed by Structures in Domains II and V of 23S Ribosomal RNA.” Molecular Microbiology 31, no. 2: 623–631. 10.1046/J.1365-2958.1999.01202.X. 10027978

[emi70097-bib-0065] Haslbeck, M. , T. Franzmann , D. Weinfurtner , and J. Buchner . 2005. “Some Like It Hot: The Structure and Function of Small Heat‐Shock Proteins.” Nature Structural & Molecular Biology 12, no. 10: 842–846. 10.1038/nsmb993. 16205709

[emi70097-bib-0066] Hooper, D. C. , and G. A. Jacoby . 2015. “Mechanisms of Drug Resistance: Quinolone Resistance.” Annals of the New York Academy of Sciences 1354, no. 1: 12–31. 10.1111/NYAS.12830. 26190223 PMC4626314

[emi70097-bib-0067] Hyatt, D. , G. L. Chen , P. F. LoCascio , M. L. Land , F. W. Larimer , and L. J. Hauser . 2010. “Prodigal: Prokaryotic Gene Recognition and Translation Initiation Site Identification.” BMC Bioinformatics 11, no. 1: 1–11. 10.1186/1471-2105-11-119/TABLES/5. 20211023 PMC2848648

[emi70097-bib-0068] Itoh, T. , N. Yoshikawa , and T. Takashina . 2007. “ *Thermogymnomonas acidicola* Gen. Nov., Sp. Nov., a Novel Thermoacidophilic, Cell Wall‐Less Archaeon in the Order Thermoplasmatales, Isolated From a Solfataric Soil in Hakone, Japan.” International Journal of Systematic and Evolutionary Microbiology 57, no. Pt 11: 2557–2561. 10.1099/IJS.0.65203-0. 17978217

[emi70097-bib-0069] Ivanova, A. A. , K. K. Miroshnikov , and I. Y. Oshkin . 2021. “Exploring Antibiotic Susceptibility, Resistome and Mobilome Structure of planctomycetes From Gemmataceae Family.” Sustainability (Switzerland) 13, no. 9: 5031. 10.3390/SU13095031/S1.

[emi70097-bib-0070] James, P. D. A. , and C. Edwards . 1989. “The Effects of Temperature on Growth and Production of the Antibiotic Granaticin by a Thermotolerant Streptomycete.” Journal of General Microbiology 135, no. 7: 1997–2003. 10.1099/00221287-135-7-1997/CITE/REFWORKS. 2575655

[emi70097-bib-0071] Jardetzky, O. 1963. “Studies on the Mechanism of Action of Chloramphenicol: I. The Conformation of Chloramphenicol in Solution.” Journal of Biological Chemistry 238, no. 7: 2498–2508. 10.1016/S0021-9258(19)68000-2. 13957484

[emi70097-bib-0072] Johnston, N. , T. Mukhtar , and G. Wright . 2005. “Streptogramin Antibiotics: Mode of Action and Resistance.” Current Drug Targets 3, no. 4: 335–344. 10.2174/1389450023347678. 12102603

[emi70097-bib-0073] Kaba, H. E. J. , E. Kuhlmann , and S. Scheithauer . 2020. “Thinking Outside the Box: Association of Antimicrobial Resistance With Climate Warming in Europe – A 30 Country Observational Study.” International Journal of Hygiene and Environmental Health 223, no. 1: 151–158. 10.1016/J.IJHEH.2019.09.008. 31648934

[emi70097-bib-0074] Kamal, S. M. , D. J. Simpson , Z. Wang , M. Gänzle , and U. Römling . 2021. “Horizontal Transmission of Stress Resistance Genes Shape the Ecology of Beta‐ and Gamma‐Proteobacteria.” Frontiers in Microbiology 12: 1758. 10.3389/FMICB.2021.696522/BIBTEX. PMC8290217 34295324

[emi70097-bib-0075] Kappé, G. , J. A. M. Leunissen , and W. W. de Jong . 2002. “Evolution and Diversity of Prokaryotic Small Heat Shock Proteins.” Progress in Molecular and Subcellular Biology 28: 1–17. 10.1007/978-3-642-56348-5_1. 11908054

[emi70097-bib-0076] Kester, J. C. , and S. M. Fortune . 2014. “Persisters and Beyond: Mechanisms of Phenotypic Drug Resistance and Drug Tolerance in Bacteria.” Critical Reviews in Biochemistry and Molecular Biology 49, no. 2: 91–101. 10.3109/10409238.2013.869543. 24328927

[emi70097-bib-0077] Kohanski, M. A. , D. J. Dwyer , J. Wierzbowski , G. Cottarel , and J. J. Collins . 2008. “Mistranslation of Membrane Proteins and Two‐Component System Activation Trigger Antibiotic‐Mediated Cell Death.” Cell 135, no. 4: 679–690. 10.1016/J.CELL.2008.09.038/ATTACHMENT/A23BBD22-97F2-4C70-9F05-3DC72588A070/MMC5.XLS. 19013277 PMC2684502

[emi70097-bib-0078] Kong, F. , Z. Qi , H. Tong , N. Ren , and S. You . 2024. “Case Study on the Relationship Between Transmission of Antibiotic Resistance Genes and Microbial Community Under Freeze‐Thaw Cycle on Cold‐Region Dairy Farm.” Science of the Total Environment 952: 175989. 10.1016/J.SCITOTENV.2024.175989. 39233087

[emi70097-bib-0079] Kong, H. H. , J. Oh , C. Deming , et al. 2012. “Temporal Shifts in the Skin Microbiome Associated With Disease Flares and Treatment in Children With Atopic Dermatitis.” Genome Research 22, no. 5: 850–859. 10.1101/GR.131029.111. 22310478 PMC3337431

[emi70097-bib-0080] Kopylova, E. , L. Noé , and H. Touzet . 2012. “SortMeRNA: Fast and Accurate Filtering of Ribosomal RNAs in Metatranscriptomic Data.” Bioinformatics 28, no. 24: 3211–3217. 10.1093/BIOINFORMATICS/BTS611. 23071270

[emi70097-bib-0081] Kuykendall, L. D. , M. A. Roy , J. J. O'Neill , and T. E. Devine . 1988. “Fatty Acids, Antibiotic Resistance, and Deoxyribonucleic Acid Homology Groups of *Bradyrhizobium japonicum* .” International Journal of Systematic Bacteriology 38, no. 4: 358–361. 10.1099/00207713-38-4-358/CITE/REFWORKS.

[emi70097-bib-0082] Ladau, J. , Y. Shi , X. Jing , et al. 2018. “Existing Climate Change Will Lead to Pronounced Shifts in the Diversity of Soil Prokaryotes.” MSystems 3, no. 5: 1–15. 10.1128/msystems.00167-18. PMC6199470 30374458

[emi70097-bib-0083] Lakens, D. 2013. “Calculating and Reporting Effect Sizes to Facilitate Cumulative Science: A Practical Primer for T‐Tests and ANOVAs.” Frontiers in Psychology 4, no. 863: 1–12. 10.3389/FPSYG.2013.00863/ABSTRACT. 24324449 PMC3840331

[emi70097-bib-0084] Layton, J. C. , and P. L. Foster . 2005. “Error‐Prone DNA Polymerase IV is Regulated by the Heat Shock Chaperone GroE in *Escherichia coli* .” Journal of Bacteriology 187, no. 2: 449. 10.1128/JB.187.2.449-457.2005. 15629916 PMC543561

[emi70097-bib-0085] Levy, S. B. , and M. Bonnie . 2004. “Antibacterial Resistance Worldwide: Causes, Challenges and Responses.” Nature Medicine 10, no. 12: S122–S129. 10.1038/nm1145. 15577930

[emi70097-bib-0086] Li, D. , C. M. Liu , R. Luo , K. Sadakane , and T. W. Lam . 2015. “MEGAHIT: An Ultra‐Fast Single‐Node Solution for Large and Complex Metagenomics Assembly via Succinct de Bruijn Graph.” Bioinformatics (Oxford, England) 31, no. 10: 1674–1676. 10.1093/BIOINFORMATICS/BTV033. 25609793

[emi70097-bib-0087] Li, D. , M. Yang , J. Hu , et al. 2009. “Antibiotic‐Resistance Profile in Environmental Bacteria Isolated From Penicillin Production Wastewater Treatment Plant and the Receiving River.” Environmental Microbiology 11, no. 6: 1506–1517. 10.1111/J.1462-2920.2009.01878.X. 19226301

[emi70097-bib-0088] Li, H. B. , A. M. Hou , T. J. Chen , et al. 2021. “Decreased Antibiotic Susceptibility in *Pseudomonas aeruginosa* Surviving UV Irradition.” Frontiers in Microbiology 12: 604245. 10.3389/FMICB.2021.604245/BIBTEX. 33613479 PMC7886673

[emi70097-bib-0089] Li, W. , C. Liu , H. C. Ho , et al. 2022. “Association Between Antibiotic Resistance and Increasing Ambient Temperature in China: An Ecological Study With Nationwide Panel Data.” Lancet Regional Health – Western Pacific 30: 1–11. 10.1016/J.LANWPC.2022.100628. PMC9672962 36406382

[emi70097-bib-0090] Li, W. , C. Liu , H. C. Ho , et al. 2023. “Estimating the Effect of Increasing Ambient Temperature on Antimicrobial Resistance in China: A Nationwide Ecological Study With the Difference‐in‐Differences Approach.” Science of the Total Environment 882: 1–7. 10.1016/J.SCITOTENV.2023.163518. 37080321

[emi70097-bib-0091] Li, Z. , A. Sun , X. Liu , et al. 2022. “Climate Warming Increases the Proportions of Specific Antibiotic Resistance Genes in Natural Soil Ecosystems.” Journal of Hazardous Materials 430: 128442. 10.1016/J.JHAZMAT.2022.128442. 35158246

[emi70097-bib-0092] Lindquist, S. 1986. “The Heat‐Shock Response.” Annual Review of Biochemistry 55: 1151–1191. 10.1146/ANNUREV.BI.55.070186.005443. 2427013

[emi70097-bib-0093] Loughman, K. , J. Hall , S. Knowlton , et al. 2016. “Temperature‐Dependent Gentamicin Resistance of *Francisella tularensis* is Mediated by Uptake Modulation.” Frontiers in Microbiology 7: 178367. 10.3389/FMICB.2016.00037/BIBTEX. PMC4729955 26858709

[emi70097-bib-0094] Love, M. I. , W. Huber , and S. Anders . 2014. “Moderated Estimation of Fold Change and Dispersion for RNA‐Seq Data With DESeq2.” Genome Biology 15, no. 12: 1–21. 10.1186/S13059-014-0550-8/FIGURES/9. PMC4302049 25516281

[emi70097-bib-0095] Lovero, K. G. , and L. Mota‐Bravo . 2022. “Closed Genome Sequence of an Environmental *Aeromonas veronii* Strain From California, United States, With an IncA/C Plasmid Carrying an Extended‐Spectrum β‐Lactamase Gene, Bla VEB‐3.” Microbiology Resource Announcements 11, no. 3: 1–4. 10.1128/mra.01033-21. PMC8928778 35195453

[emi70097-bib-0096] Łupkowska, A. , S. Monem , J. Dębski , K. Stojowska‐Swędrzyńska , D. Kuczyńska‐Wiśnik , and E. Laskowska . 2023. “Protein Aggregation and Glycation in *Escherichia coli* Exposed to Desiccation‐Rehydration Stress.” Microbiological Research 270: 127335. 10.1016/J.MICRES.2023.127335. 36841129

[emi70097-bib-0097] MacFadden, D. R. , S. F. McGough , D. Fisman , M. Santillana , and J. S. Brownstein . 2018. “Antibiotic Resistance Increases With Local Temperature.” Nature Climate Change 8, no. 6: 510–514. 10.1038/s41558-018-0161-6. PMC6201249 30369964

[emi70097-bib-0098] Mackey, B. M. , C. A. Miles , S. E. Parsons , and D. A. Seymour . 1991. “Thermal Denaturation of Whole Cells and Cell Components of *Escherichia coli* Examined by Differential Scanning Calorimetry.” Journal of General Microbiology 137, no. 10: 2361–2374. 10.1099/00221287-137-10-2361/CITE/REFWORKS. 1722814

[emi70097-bib-0099] MacLean, M. J. , L. S. Ness , G. P. Ferguson , and I. R. Booth . 1998. “The Role of Glyoxalase I in the Detoxification of Methylglyoxal and in the Activation of the KefB K + Efflux System in *Escherichia coli* .” Molecular Microbiology 27, no. 3: 563–571. 10.1046/J.1365-2958.1998.00701.X. 9489668

[emi70097-bib-0100] MacLean, R. C. , C. Torres‐Barceló , and R. Moxon . 2013. “Evaluating Evolutionary Models of Stress‐Induced Mutagenesis in Bacteria.” Nature Reviews Genetics 14, no. 3: 221–227. 10.1038/nrg3415. 23400102

[emi70097-bib-0101] Maki, Y. , and H. Yoshida . 2021. “Ribosomal Hibernation‐Associated Factors in *Escherichia coli* .” Microorganisms 10, no. 1: 1–11. 10.3390/MICROORGANISMS10010033. 35056482 PMC8778775

[emi70097-bib-0102] Marti, R. , M. Muniesa , M. Schmid , C. H. Ahrens , J. Naskova , and J. Hummerjohann . 2016. “Short Communication: Heat‐Resistant *Escherichia coli* as Potential Persistent Reservoir of Extended‐Spectrum β‐Lactamases and Shiga Toxin‐Encoding Phages in Dairy.” Journal of Dairy Science 99, no. 11: 8622–8632. 10.3168/JDS.2016-11076. 27568050

[emi70097-bib-0103] Martinez, J. L. 2009. “Environmental Pollution by Antibiotics and by Antibiotic Resistance Determinants.” Environmental Pollution (Barking, Essex: 1987) 157, no. 11: 2893–2902. 10.1016/J.ENVPOL.2009.05.051. 19560847

[emi70097-bib-0104] Martínez, J. L. , and F. Rojo . 2011. “Metabolic Regulation of Antibiotic Resistance.” FEMS Microbiology Reviews 35, no. 5: 768–789. 10.1111/J.1574-6976.2011.00282.X. 21645016

[emi70097-bib-0105] Martiny, A. C. , J. B. H. Martiny , C. Weihe , A. Field , and J. C. Ellis . 2011. “Functional Metagenomics Reveals Previously Unrecognized Diversity of Antibiotic Resistance Genes in Gulls.” Frontiers in Microbiology 2: 238. 10.3389/FMICB.2011.00238/BIBTEX. 22347872 PMC3275322

[emi70097-bib-0106] McGough, S. F. , D. R. MacFadden , M. W. Hattab , K. Mølbak , and M. Santillana . 2020. “Rates of Increase of Antibiotic Resistance and Ambient Temperature in Europe: A Cross‐National Analysis of 28 Countries Between 2000 and 2016.” Eurosurveillance 25, no. 45: 1–12. 10.2807/1560-7917.ES.2020.25.45.1900414. PMC7667635 33183408

[emi70097-bib-0107] McKew, B. A. , J. D. Taylor , T. J. McGenity , and G. J. C. Underwood . 2011. “Resistance and Resilience of Benthic Biofilm Communities From a Temperate Saltmarsh to Desiccation and Rewetting.” ISME Journal 5, no. 1: 30–41. 10.1038/ISMEJ.2010.91. 20596071 PMC3105671

[emi70097-bib-0108] Morar, M. , K. Bhullar , D. W. Hughes , M. Junop , and G. D. Wright . 2009. “Structure and Mechanism of the Lincosamide Antibiotic Adenylyltransferase LinB.” Structure 17, no. 12: 1649–1659. 10.1016/J.STR.2009.10.013. 20004168

[emi70097-bib-0109] Mueller, E. A. , and P. A. Levin . 2020. “Bacterial Cell Wall Quality Control During Environmental Stress.” MBio 11, no. 5: e02456‐20. 10.1128/MBIO.02456-20. 33051371 PMC7554673

[emi70097-bib-0110] Mueller, J. G. , H. D. Skipper , E. R. Shipe , L. W. Grimes , and S. C. Wagner . 1988. “Intrinsic Antibiotic Resistance in *Bradyrhizobium japonicum* .” Soil Biology and Biochemistry 20, no. 6: 879–882. 10.1016/0038-0717(88)90097-1.

[emi70097-bib-0111] Mullis, M. M. , I. M. Rambo , B. J. Baker , and B. K. Reese . 2019. “Diversity, Ecology, and Prevalence of Antimicrobials in Nature.” Frontiers in Microbiology 10: 482299. 10.3389/fmicb.2019.02518. PMC6869823 31803148

[emi70097-bib-0112] Nascimento, A. A. , L. Cursino , H. Gonç Alves‐dornelas , A. Reis , E. Chartone‐Souza , and M. A. Marini . 2003. “Antibiotic‐Resistant Gram‐Negative Bacteria in Birds From the Brazilian Atlantic Forest.” Condor 105, no. 2: 358–361. 10.1093/CONDOR/105.2.358.

[emi70097-bib-0113] Nash, K. A. , N. Andini , Y. Zhang , B. A. Brown‐Elliott , and R. J. Wallace . 2006. “Intrinsic Macrolide Resistance in Rapidly Growing Mycobacteria.” Antimicrobial Agents and Chemotherapy 50, no. 10: 3476. 10.1128/AAC.00402-06. 17005837 PMC1610084

[emi70097-bib-0114] Nguyen, C. C. , C. N. Hugie , M. L. Kile , and T. Navab‐Daneshmand . 2019. “Association Between Heavy Metals and Antibiotic‐Resistant Human Pathogens in Environmental Reservoirs: A Review.” Frontiers of Environmental Science & Engineering 13, no. 3: 1–17. 10.1007/S11783-019-1129-0/METRICS.

[emi70097-bib-0115] Njenga, R. , J. Boele , Y. Öztürk , and H. G. Koch . 2023. “Coping With Stress: How Bacteria Fine‐Tune Protein Synthesis and Protein Transport.” Journal of Biological Chemistry 299, no. 9: 105163. 10.1016/J.JBC.2023.105163. 37586589 PMC10502375

[emi70097-bib-0116] Nolivos, S. , J. Cayron , A. Dedieu , A. Page , F. Delolme , and C. Lesterlin . 2019. “Role of AcrAB‐TolC Multidrug Efflux Pump in Drug‐Resistance Acquisition by Plasmid Transfer.” Science (New York, N.Y.) 364, no. 6442: 778–782. 10.1126/SCIENCE.AAV6390. 31123134

[emi70097-bib-0117] Ohno, A. , T. Ano , and M. Shoda . 1995. “Effect of Temperature on Production of Lipopeptide Antibiotics, Iturin A and Surfactin by a Dual Producer, *Bacillus subtilis* RB14, in Solid‐State Fermentation.” Journal of Fermentation and Bioengineering 80, no. 5: 517–519. 10.1016/0922-338X(96)80930-5. 18623394

[emi70097-bib-0118] Olson, R. D. , R. Assaf , T. Brettin , et al. 2023. “Introducing the Bacterial and Viral Bioinformatics Resource Center (BV‐BRC): A Resource Combining PATRIC, IRD and ViPR.” Nucleic Acids Research 51, no. 1D: 678–689. 10.1093/nar/gkac1003. PMC9825582 36350631

[emi70097-bib-0119] Pärnänen, K. M. M. , C. Narciso‐Da‐Rocha , D. Kneis , et al. 2019. “Antibiotic Resistance in European Wastewater Treatment Plants Mirrors the Pattern of Clinical Antibiotic Resistance Prevalence.” Science Advances 5, no. 3: 1–10. 10.1126/SCIADV.AAU9124. PMC6436925 30944853

[emi70097-bib-0120] Patro, R. , G. Duggal , and C. Kingsford . 2018. “Salmon: Accurate, Versatile and Ultrafast Quantification From RNA‐Seq Data Using Lightweight‐Alignment.” 10.1184/R1/6702233.V1.

[emi70097-bib-0121] Pham, T. D. M. , Z. M. Ziora , and M. A. T. Blaskovich . 2019. “Quinolone Antibiotics.” MedChemComm 10, no. 10: 1719–1739. 10.1039/C9MD00120D. 31803393 PMC6836748

[emi70097-bib-0122] Poole, K. 2014. “Stress Responses as Determinants of Antimicrobial Resistance in *Pseudomonas aeruginosa* : Multidrug Efflux and more1.” Canadian Journal of Microbiology 60, no. 12: 783–791. 10.1139/CJM-2014-0666. 25388098

[emi70097-bib-0123] Potts, M. 1994. “Desiccation Tolerance of Prokaryotes.” Microbiological Reviews 58, no. 4: 755–805. 10.1128/MR.58.4.755-805.1994. 7854254 PMC372989

[emi70097-bib-0124] R Core Team . 2013. A Language and Environment for Statistical Computing. R Foundation for Statistical Computing.

[emi70097-bib-0125] Raaijmakers, J. M. , M. Vlami , and J. T. de Souza . 2002. “Antibiotic Production by Bacterial Biocontrol Agents.” Antonie van Leeuwenhoek International Journal of General and Molecular Microbiology 81, no. 1–4: 537–547. 10.1023/A:1020501420831/METRICS. 12448749

[emi70097-bib-0126] Rahmati‐bahram, A. , J. T. Magee , and S. K. Jackson . 1995. “Growth Temperature‐Dependent Variation of Cell Envelope Lipids and Antibiotic Susceptibility in Stenotrophomonas (Xanthomonas) Maltophilia.” Journal of Antimicrobial Chemotherapy 36, no. 2: 317–326. 10.1093/JAC/36.2.317. 8522461

[emi70097-bib-0127] Ratkowsky, D. A. , J. Olley , T. A. McMeekin , and A. Ball . 1982. “Relationship Between Temperature and Growth Rate of Bacterial Cultures.” Journal of Bacteriology 149, no. 1: 1. 10.1128/JB.149.1.1-5.1982. 7054139 PMC216584

[emi70097-bib-0128] Richter, K. , M. Haslbeck , and J. Buchner . 2010. “The Heat Shock Response: Life on the Verge of Death.” Molecular Cell 40, no. 2: 253–266. 10.1016/J.MOLCEL.2010.10.006. 20965420

[emi70097-bib-0129] Roberts, M. C. 2005. “Update on Acquired Tetracycline Resistance Genes.” FEMS Microbiology Letters 245, no. 2: 195–203. 10.1016/J.FEMSLE.2005.02.034. 15837373

[emi70097-bib-0130] Roberts, M. C. 2011. “Environmental Macrolide‐Lincosamide‐Streptogramin and Tetracycline Resistant Bacteria.” Frontiers in Microbiology 2: 9102. 10.3389/FMICB.2011.00040/BIBTEX. PMC3153021 21833302

[emi70097-bib-0131] Roberts, M. C. , and S. Schwarz . 2016. “Tetracycline and Phenicol Resistance Genes and Mechanisms: Importance for Agriculture, the Environment, and Humans.” Journal of Environmental Quality 45, no. 2: 576–592. 10.2134/JEQ2015.04.0207. 27065405

[emi70097-bib-0132] Rodríguez‐Verdugo, A. , B. S. Gaut , and O. Tenaillon . 2013. “Evolution of *Escherichia coli* Rifampicin Resistance in an Antibiotic‐Free Environment During Thermal Stress.” BMC Evolutionary Biology 13, no. 1: 1–11. 10.1186/1471-2148-13-50/TABLES/3. 23433244 PMC3598500

[emi70097-bib-0133] Rodríguez‐Verdugo, A. , N. Lozano‐Huntelman , M. Cruz‐Loya , V. Savage , and P. Yeh . 2020. “Compounding Effects of Climate Warming and Antibiotic Resistance.” iScience 23, no. 4: 101024. 10.1016/J.ISCI.2020.101024. 32299057 PMC7160571

[emi70097-bib-0134] Romero‐Olivares, A. L. , G. Meléndrez‐Carballo , A. Lago‐Lestón , and K. K. Treseder . 2019. “Soil Metatranscriptomes Under Long‐Term Experimental Warming and Drying: Fungi Allocate Resources to Cell Metabolic Maintenance Rather Than Decay.” Frontiers in Microbiology 10: 1914. 10.3389/FMICB.2019.01914/BIBTEX. 31551941 PMC6736569

[emi70097-bib-0135] Romero‐Olivares, A. L. , J. W. Taylor , and K. K. Treseder . 2015. “Neurospora Discreta as a Model to Assess Adaptation of Soil Fungi to Warming.” BMC Evolutionary Biology 15, no. 1: 198. 10.1186/s12862-015-0482-2. 26377599 PMC4573461

[emi70097-bib-0136] Rousk, J. , S. D. Frey , and E. Bååth . 2012. “Temperature Adaptation of Bacterial Communities in Experimentally Warmed Forest Soils.” Global Change Biology 18, no. 10: 3252–3258. 10.1111/J.1365-2486.2012.02764.X. 28741822

[emi70097-bib-0137] Saeki, Y. , I. Akagi , H. Takaki , and Y. Nagatomo . 2012. “Diversity of Indigenous Bradyrhizobium Strains Isolated From Three Different Rj‐Soybean Cultivars in Terms of Randomly Amplified Polymorphic DNA and Intrinsic Antibiotic Resistance.” Soil Science and Plant Nutrition 46, no. 4: 917–926. 10.1080/00380768.2000.10409157.

[emi70097-bib-0138] Santarella‐Mellwig, R. , S. Pruggnaller , N. Roos , I. W. Mattaj , and D. P. Devos . 2013. “Three‐Dimensional Reconstruction of Bacteria With a Complex Endomembrane System.” PLoS Biology 11, no. 5: e1001565. 10.1371/JOURNAL.PBIO.1001565. 23700385 PMC3660258

[emi70097-bib-0139] Sayah, R. S. , J. B. Kaneene , Y. Johnson , and R. A. Miller . 2005. “Patterns of Antimicrobial Resistance Observed in *Escherichia coli* Isolates Obtained From Domestic‐ and Wild‐Animal Fecal Samples, Human Septage, and Surface Water.” Applied and Environmental Microbiology 71, no. 3: 1394–1404. 10.1128/AEM.71.3.1394-1404.2005. 15746342 PMC1065171

[emi70097-bib-0140] Schages, L. , F. Wichern , R. Kalscheuer , and D. Bockmühl . 2020. “Winter is Coming – Impact of Temperature on the Variation of Beta‐Lactamase and Mcr Genes in a Wastewater Treatment Plant.” Science of the Total Environment 712: 136499. 10.1016/J.SCITOTENV.2020.136499. 31945531

[emi70097-bib-0141] Schlünzen, F. , R. Zarivach , J. Harms , et al. 2001. “Structural Basis for the Interaction of Antibiotics With the Peptidyl Transferase Centre in Eubacteria.” Nature 413, no. 6858: 814–821. 10.1038/35101544. 11677599

[emi70097-bib-0142] Schramm, F. D. , K. Schroeder , and K. Jonas . 2020. “Protein Aggregation in Bacteria.” FEMS Microbiology Reviews 44, no. 1: 54–72. 10.1093/FEMSRE/FUZ026. 31633151 PMC7053576

[emi70097-bib-0143] Schwartzman, D. W. , and C. H. Lineweaver . 2004. “The Hyperthermophilic Origin of Life Revisited.” Biochemical Society Transactions 32, no. 2: 168–171. 10.1042/BST0320168. 15046564

[emi70097-bib-0144] Seiler, C. , and T. U. Berendonk . 2012. “Heavy Metal Driven Co‐Selection of Antibiotic Resistance in Soil and Water Bodies Impacted by Agriculture and Aquaculture.” Frontiers in Microbiology 3: 399. 10.3389/FMICB.2012.00399/BIBTEX. 23248620 PMC3522115

[emi70097-bib-0145] Seyfried, E. E. , R. J. Newton , K. F. Rubert IV , J. A. Pedersen , and K. D. McMahon . 2010. “Occurrence of Tetracycline Resistance Genes in Aquaculture Facilities With Varying Use of Oxytetracycline.” Microbial Ecology 59, no. 4: 799–807. 10.1007/S00248-009-9624-7. 20217406 PMC4066850

[emi70097-bib-0146] Somorin, Y. , F. Abram , F. Brennan , and C. O'Byrne . 2016. “The General Stress Response is Conserved in Long‐Term Soil‐Persistent Strains of *Escherichia coli* .” Applied and Environmental Microbiology 82, no. 15: 4628–4640. 10.1128/AEM.01175-16. 27235429 PMC4984288

[emi70097-bib-0147] Spížek, J. , and T. Řezanka . 2017. “Lincosamides: Chemical Structure, Biosynthesis, Mechanism of Action, Resistance, and Applications.” Biochemical Pharmacology 133: 20–28. 10.1016/j.bcp.2016.12.001. 27940264

[emi70097-bib-0148] Starosta, A. L. , J. Lassak , K. Jung , and D. N. Wilson . 2014. “The Bacterial Translation Stress Response.” FEMS Microbiology Reviews 38, no. 6: 1172–1201. 10.1111/1574-6976.12083. 25135187 PMC4227928

[emi70097-bib-0149] Stetter, K. O. 2006. “Hyperthermophiles in the History of Life.” Philosophical Transactions of the Royal Society, B: Biological Sciences 361, no. 1474: 1837. 10.1098/RSTB.2006.1907. PMC1664684 17008222

[emi70097-bib-0150] Sutcliffe, J. A. , and R. Leclercq . 2002. “Mechanisms of Resistance to Macrolides, Lincosamides, and Ketolides.” In Macrolide Antibiotics, 281–317. Birkhäuser. 10.1007/978-3-0348-8105-0_17.

[emi70097-bib-0151] Święciło, A. 2016. “Cross‐Stress Resistance in *Saccharomyces cerevisiae* Yeast—New Insight Into an Old Phenomenon.” Cell Stress & Chaperones 21, no. 2: 187–200. 10.1007/S12192-016-0667-7. 26825800 PMC4786536

[emi70097-bib-0152] Teixeira, P. , H. Castro , C. Mohácsi‐Farkas , and R. Kirby . 1997. “Identification of Sites of Injury in *Lactobacillus bulgaricus* During Heat Stress.” Journal of Applied Microbiology 83, no. 2: 219–226. 10.1046/J.1365-2672.1997.00221.X. 9281825

[emi70097-bib-0153] Tenson, T. , M. Lovmar , and M. Ehrenberg . 2003. “The Mechanism of Action of Macrolides, Lincosamides and Streptogramin B Reveals the Nascent Peptide Exit Path in the Ribosome.” Journal of Molecular Biology 330, no. 5: 1005–1014. 10.1016/S0022-2836(03)00662-4. 12860123

[emi70097-bib-0154] Thackray, P. D. , and A. Moir . 2003. “SigM, an Extracytoplasmic Function Sigma Factor of *Bacillus subtilis* , Is Activated in Response to Cell Wall Antibiotics, Ethanol, Heat, Acid, and Superoxide Stress.” Journal of Bacteriology 185, no. 12: 3491–3498. 10.1128/JB.185.12.3491-3498.2003/ASSET/F262F8DA-189F-4669-9548-9C88005EAB79/ASSETS/GRAPHIC/JB1231306006.JPEG. 12775685 PMC156226

[emi70097-bib-0155] Tiwari, K. , and R. K. Gupta . 2012. “Rare Actinomycetes: A Potential Storehouse for Novel Antibiotics.” Critical Reviews in Biotechnology 32, no. 2: 108–132. 10.3109/07388551.2011.562482. 21619453

[emi70097-bib-0156] Toledo, S. L. O. , R. M. S. Silva , I. C. R. dos Santos , W. G. Lima , L. G. R. Ferreira , and M. C. Paiva . 2020. “Domestic Wastewater Treatment Plants as Sources of Macrolide‐Lincosamide‐Streptogramin B and Penicillin‐Resistant *Staphylococcus aureus* in Brazil.” Revista Colombiana de Ciencias Quimico‐Farmaceuticas (Colombia) 49, no. 2: 267–279. 10.15446/RCCIQUIFA.V49N2.88854.

[emi70097-bib-0157] Tonyali, B. , A. McDaniel , V. Trinetta , and U. Yucel . 2019. “Evaluation of Heating Effects on the Morphology and Membrane Structure of *Escherichia coli* Using electron Paramagnetic Resonance Spectroscopy.” Biophysical Chemistry 252: 106191. 10.1016/J.BPC.2019.106191. 31177024

[emi70097-bib-0158] Treseder, K. K. , M. C. Mack , and A. Cross . 2004. “Relationships Among Fires, Fungi, and Soil Dynamics in Alaskan Boreal Forests.” Ecological Applications 14, no. 6: 1826–1838. 10.1890/03-5133.

[emi70097-bib-0159] Usary, J. , and W. S. Champney . 2001. “Erythromycin Inhibition of 50S Ribosomal Subunit Formation in *Escherichia coli* Cells.” Molecular Microbiology 40, no. 4: 951–962. 10.1046/J.1365-2958.2001.02438.X. 11401702

[emi70097-bib-0160] VanBogelen, R. A. , and F. C. Neidhardt . 1990. “Ribosomes as Sensors of Heat and Cold Shock in *Escherichia coli* .” Proceedings of the National Academy of Sciences of the United States of America 87, no. 15: 5589–5593. 10.1073/PNAS.87.15.5589. 2198567 PMC54372

[emi70097-bib-0161] Vannuffel, P. , and C. Cocito . 1996. “Mechanism of Action of Streptogramins and Macrolides.” Drugs 51: 20–30. 10.2165/00003495-199600511-00006/METRICS. 8724813

[emi70097-bib-0162] Venglarcik, J. S. , L. L. Blair , and L. M. Dunkle . 1983. “pH‐Dependent Oxacillin Tolerance of *Staphylococcus aureus* .” Antimicrobial Agents and Chemotherapy 23, no. 2: 232–235. 10.1128/AAC.23.2.232. 6551162 PMC186027

[emi70097-bib-0163] Wagner, I. D. , and J. Wiegel . 2008. “Diversity of Thermophilic Anaerobes.” Annals of the New York Academy of Sciences 1125, no. 1: 1–43. 10.1196/ANNALS.1419.029. 18378585

[emi70097-bib-0164] Waksman, S. A. , and F. C. Gerretsen . 1931. “Influence of Temperature and Moisture Upon the Nature and Extent of Decomposition of Plant Residues by Microorganisms.” Ecology 12, no. 1: 33–60. 10.2307/1932933.

[emi70097-bib-0165] Walsh, T. R. , J. Weeks , D. M. Livermore , and M. A. Toleman . 2011. “Dissemination of NDM‐1 Positive bacteria in the New Delhi Environment and Its Implications for Human Health: An Environmental Point Prevalence Study.” Lancet Infectious Diseases 11, no. 5: 355–362. 10.1016/S1473-3099(11)70059-7. 21478057

[emi70097-bib-0166] Wang, T. , C. Liang , M. Zheng , et al. 2020. “Ribosome Hibernation as a Stress Response of Bacteria.” Protein & Peptide Letters 27, no. 11: 1082–1091. 10.2174/0929866527666200610142118. 32520673

[emi70097-bib-0167] Wang, Z. , Q. Chen , J. Zhang , et al. 2024. “Climate Warming Promotes Collateral Antibiotic Resistance Development in Cyanobacteria.” Water Research 256: 121642. 10.1016/J.WATRES.2024.121642. 38657307

[emi70097-bib-0168] Wang, Z. , Y. Fang , S. Zhi , et al. 2020. “The Locus of Heat Resistance Confers Resistance to Chlorine and Other Oxidizing Chemicals in *Escherichia coli* .” Applied and Environmental Microbiology 86, no. 4: 1–16. 10.1128/AEM.02123-19. PMC6997732 31811037

[emi70097-bib-0169] Wattam, A. R. , J. J. Davis , R. Assaf , et al. 2017. “Improvements to PATRIC, the All‐Bacterial Bioinformatics Database and Analysis Resource Center.” Nucleic Acids Research 45, no. D1: D535–D542. 10.1093/nar/gkw1017. 27899627 PMC5210524

[emi70097-bib-0170] Wickham, H. 2011. “ggplot2.” Wiley Interdisciplinary Reviews: Computational Statistics 3, no. 2: 180–185. 10.1002/WICS.147.

[emi70097-bib-0171] Wickham, H. , M. Averick , J. Bryan , et al. 2019. “Welcome to the Tidyverse.” Journal of Open Source Software 4, no. 43: 1686.

[emi70097-bib-0172] Wolfe, A. D. , and F. E. Hahn . 1965. “Mode of Action of Chloramphenicol. Ix. Effects of Chloramphenicol Upon a Ribosomal Amino Acid Polymerization System and Its Binding to Bacterial Ribosome.” Biochimica et Biophysica Acta 95, no. 1: 146–155. 10.1016/0005-2787(65)90219-4. 14289020

[emi70097-bib-0173] Wood, D. E. , J. Lu , and B. Langmead . 2019. “Improved Metagenomic Analysis With Kraken 2.” Genome Biology 20, no. 1: 1–13. 10.1186/S13059-019-1891-0/FIGURES/2. 31779668 PMC6883579

[emi70097-bib-0174] Yang, Y. , A. J. Ashworth , J. M. DeBruyn , et al. 2020. “Antimicrobial Resistant Gene Prevalence in Soils due to Animal Manure Deposition and Long‐Term Pasture Management.” PeerJ 8: e10258. 10.7717/PEERJ.10258/SUPP-1. 33194426 PMC7646296

[emi70097-bib-0175] Yang, Z. , T. Lan , H. Luo , et al. 2024. “Emergence and Mobilization of a Novel Lincosamide Resistance Gene Lnu(I): From Environmental Reservoirs to Pathogenic Bacteria.” Science of the Total Environment 906: 167400. 10.1016/J.SCITOTENV.2023.167400. 37769725

[emi70097-bib-0176] Yi, L. , R. Xu , X. Yuan , et al. 2024. “Heat Stress Enhances the Occurrence of Erythromycin Resistance of Enterococcus Isolates in Mice Feces.” Journal of Thermal Biology 120: 103786. 10.1016/J.JTHERBIO.2024.103786. 38428103

[emi70097-bib-0177] Yoshida, H. , and A. Wada . 2014. “The 100S Ribosome: Ribosomal Hibernation Induced by Stress.” Wiley Interdisciplinary Reviews: RNA 5, no. 5: 723–732. 10.1002/WRNA.1242. 24944100

[emi70097-bib-0178] Yura, T. , H. Nagai , and H. Mori . 1993. “Regulation of the Heat‐Shock Response in Bacteria.” Annual Review of Microbiology 47: 321–350. 10.1146/ANNUREV.MI.47.100193.001541. 7504905

[emi70097-bib-0179] Zhang, Z. , Y. Ji , D. Liu , et al. 2023. “Heat Shock Protein Inhibitors Show Synergistic Antibacterial Effects With Photodynamic Therapy on Caries‐Related Streptococci In Vitro and In Vivo.” MSphere 8, no. 2: 1–17. 10.1128/MSPHERE.00679-22/ASSET/3F140D1F-61D3-4676-8AC2-2D572419FB86/ASSETS/IMAGES/MEDIUM/MSPHERE.00679-22-F007.GIF. PMC10117063 36853046

[emi70097-bib-0180] Zhi, S. , G. Banting , Q. Li , et al. 2016. “Evidence of Naturalized Stress‐Tolerant Strains of *Escherichia coli* in Municipal Wastewater Treatment Plants.” Applied and Environmental Microbiology 82, no. 18: 5505–5518. 10.1128/AEM.00143-16. 27371583 PMC5007776

