# Supplementary material for: Warming is Associated With More Encoded Antimicrobial Resistance Genes and Transcriptions Within Five Drug Classes in Soil Bacteria: A Case Study and Synthesis
Source: Environ Microbiol. 2025 Apr 22;27(4):e70097. doi: 10.1111/1462-2920.70097 (PMC12014264; doi:10.1111/1462-2920.70097)
Supplement: Supplementary file 1 — Figure S1. Positive relationship between reported optimal growth temperature and (A) the number of encoded AMR genes normalised by the number of protein‐encoding genes (PEGs) or (B) the number of total encoded AMR genes. Each symbol represents a bacterial genome isolated from soil (n = 253 genomes). Colour represents phylum. Isolates with an optimal temperature of 37°C were removed. Figure S2. Differential expression of AMR (A) and heat resistance (B) transcripts in soil microbial communities undergoing temperature warming. The x‐axis represents log2 fold change of transcript counts in warming plots compared with controls. Plots show the 112,226 transcripts with > 50 counts between all plots. Each symbol represents one transcript. Symbols in the green region are significantly upregulated in the warmed treatment, while symbols in the blue region are significantly downregulated. Filled symbols are transcripts associated with AMR (A) and heat resistance (B). Open symbols are other transcripts. Dotted lines represent significance cut‐offs for the log10 adjusted p value and log2 fold change. [file EMI-27-e70097-s001.docx]

**
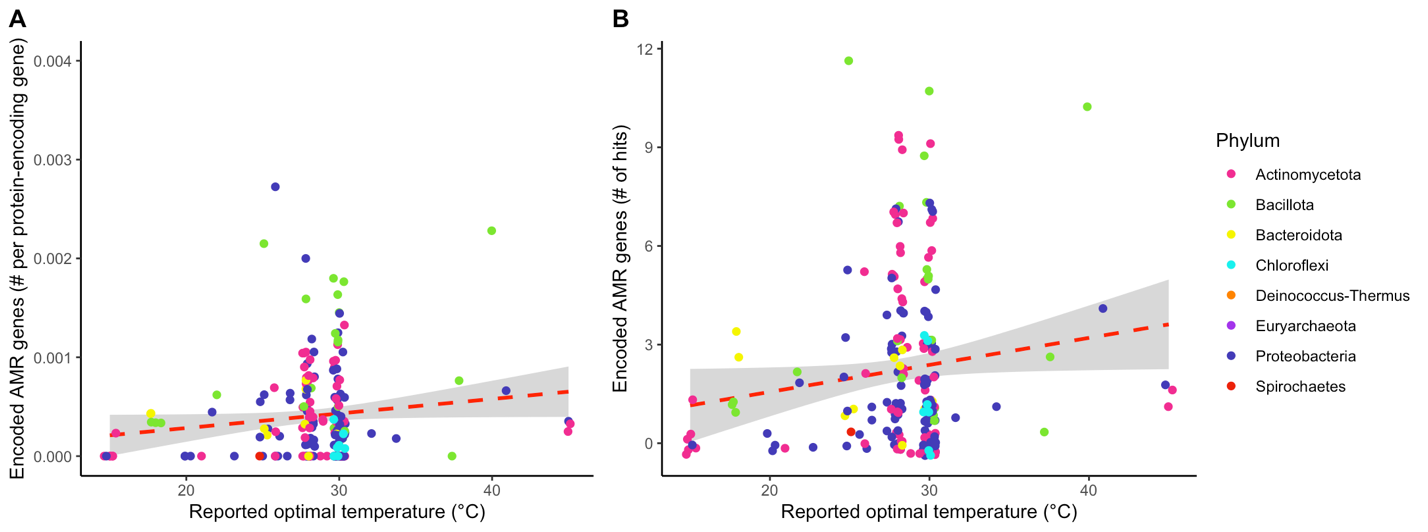
**

**Supplementary Figure 1.** Positive relationship between reported optimal growth temperature and (A) the number of encoded AMR genes normalized by the number of protein-encoding genes (PEGs) or (B) the number of total encoded AMR genes. Each symbol represents a bacterial genome isolated from soil (n = 253 genomes). Color represents phylum. Isolates with an optimal temperature of 37°C were removed.


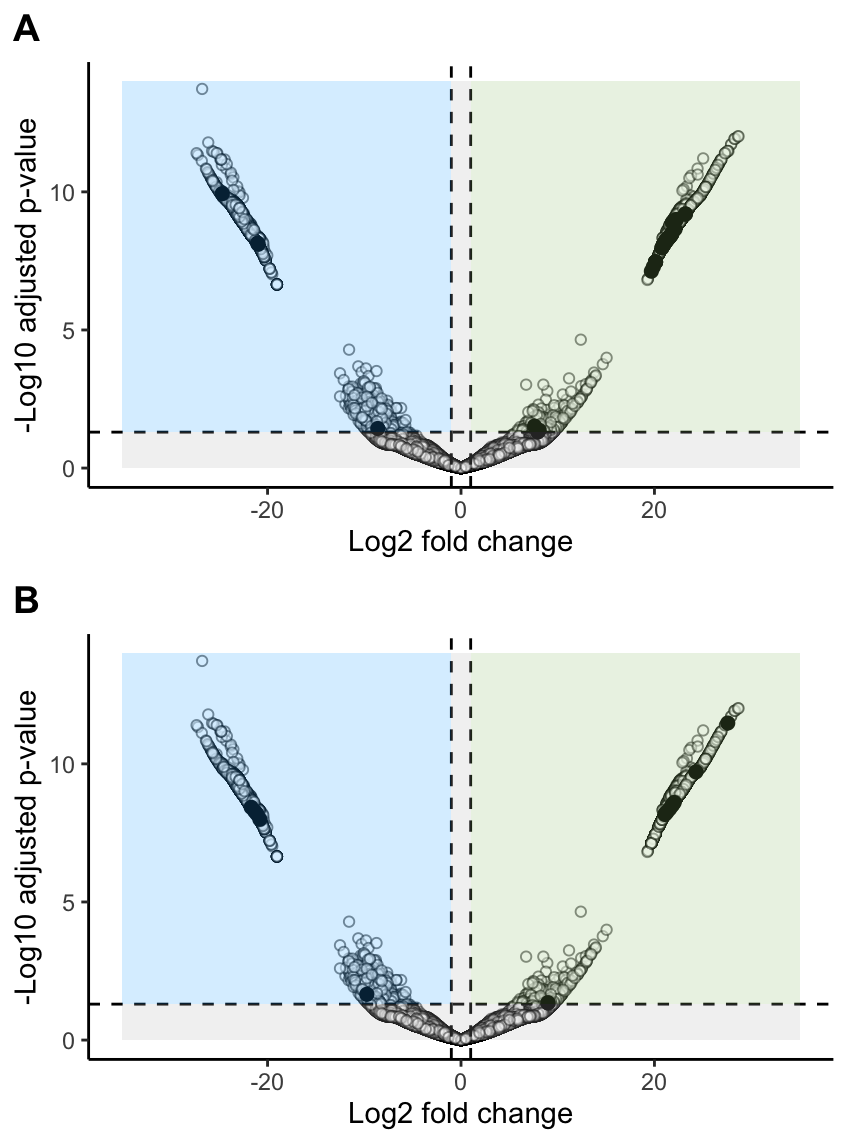


**Supplementary Figure 2.**  Differential expression of AMR (A) and heat resistance (B) transcripts in soil microbial communities undergoing temperature warming. The x-axis represents log2 fold change of transcript counts in warming plots compared to controls. Plots show the 112,226 transcripts with >50 counts between all plots. Each symbol represents one transcript. Symbols in the green region are significantly upregulated in the warmed treatment, while symbols in the blue region are significantly downregulated. Filled symbols are transcripts associated with AMR (A) and heat resistance (B). Open symbols are other transcripts. Dotted lines represent significance cut-offs for the log10 adjusted p-value and log2 fold change.
