# Supplementary material for: Warming is Associated With More Encoded Antimicrobial Resistance Genes and Transcriptions Within Five Drug Classes in Soil Bacteria: A Case Study and Synthesis
Source: Environ Microbiol. 2025 Apr 22;27(4):e70097. doi: 10.1111/1462-2920.70097 (PMC12014264; doi:10.1111/1462-2920.70097)
Supplement: Supplementary file 2 — Table S1. Significantly upregulated and downregulated heat resistance transcripts with average fold change values (p < 0.05 for all). [file EMI-27-e70097-s002.docx]

**Supplementary Table 1.** Significantly upregulated and downregulated heat resistance transcripts with average fold change values (P < 0.05 for all).

| Gene Symbol | Sequence name | Average log2 fold change |
| --- | --- | --- |
| **Upregulated** |  |  |
| shsP | Small heat shock protein sHSP20-GI | 20.88 |
| trxLHR | Heat resistance system thioredoxin Trx-GI | 21.58 |
| hsp20 | Small heat shock protein sHSP20 | 15.51 |
| kefB-GI | Heat resistance system K+/H+ antiporter KefB-G | 21.19 |
| **Downregulated** |  |  |
| shsP | Small heat shock protein sHSP20-GI | -21.07 |
| trxLHR | Heat resistance system thioredoxin Trx-GI | -17.51 |
